# 1H-Pyrrole-2,5-dicarboxylic acid, a quorum sensing inhibitor from one endophytic fungus in *Areca catechu* L., acts as antibiotic accelerant against *Pseudomonas aeruginosa*

**DOI:** 10.3389/fcimb.2024.1413728

**Published:** 2024-07-02

**Authors:** Junsheng Liu, Zhennan Wang, Yuexiang Zeng, Wei Wang, Shi Tang, Aiqun Jia

**Affiliations:** ^1^ Key Laboratory of Tropical Biological Resources of Ministry of Education, School of Pharmaceutical Sciences, Hainan University, Haikou, China; ^2^ Hainan General Hospital, Hainan Affiliated Hospital of Hainan Medical University, Haikou, China; ^3^ Modern Industrial College of Traditional Chinese Medicine and Health, Lishui University, Lishui, China

**Keywords:** *Pseudomonas aeruginosa*, 1H-pyrrole-2, 5-dicarboxylic acid, quorum sensing, virulence factors, biofilm, *Perenniporia tephropora*

## Abstract

*Pseudomonas aeruginosa* has already been stipulated as a “critical” pathogen, emphasizing the urgent need for researching and developing novel antibacterial agents due to multidrug resistance. Bacterial biofilm formation facilitates cystic fibrosis development and restricts the antibacterial potential of many current antibiotics. The capacity of *P. aeruginosa* to form biofilms and resist antibiotics is closely correlated with quorum sensing (QS). Bacterial QS is being contemplated as a promising target for developing novel antibacterial agents. QS inhibitors are a promising strategy for treating chronic infections. This study reported that the active compound PT22 (1H-pyrrole-2,5-dicarboxylic acid) isolated from *Perenniporia tephropora* FF2, one endophytic fungus from *Areca catechu* L., presents QS inhibitory activity against *P. aeruginosa*. Combined with gentamycin or piperacillin, PT22 functions as a novel antibiotic accelerant against *P. aeruginosa*. PT22 (0.50 mg/mL, 0.75 mg/mL, and 1.00 mg/mL) reduces the production of QS-related virulence factors, such as pyocyanin and rhamnolipid, and inhibits biofilm formation of *P. aeruginosa* PAO1 instead of affecting its growth. The architectural disruption of the biofilms was confirmed by visualization through scanning electron microscopy (SEM) and confocal laser scanning microscopy (CLSM). Real-time quantitative PCR (RT-qPCR) indicated that PT22 significantly attenuated the expression of QS-related genes followed by docking analysis of molecules against QS activator proteins. PT22 dramatically increased the survival rate of *Galleria mellonella*. PT22 combined with gentamycin or piperacillin presents significant inhibition of biofilm formation and eradication of mature biofilm compared to monotherapy, which was also confirmed by visualization through SEM and CLSM. After being treated with PT22 combined with gentamycin or piperacillin, the survival rates of *G. mellonella* were significantly increased compared to those of monotherapy. PT22 significantly enhanced the susceptibility of gentamycin and piperacillin against *P. aeruginosa* PAO1. Our results suggest that PT22 from *P. tephropora* FF2 as a potent QS inhibitor is a candidate antibiotic accelerant to combat the antibiotic resistance of *P. aeruginosa*.

## Introduction

Antibiotic resistance poses a critical threat to animal and human health, food security, and global environments. These mainly “ESKAPE” pathogens (*Enterococcus faecium*, *Staphylococcus aureus*, *Klebsiella pneumoniae*, *Acinetobacter baumannii*, *Pseudomonas aeruginosa*, and *Enterobacter* spp.) possess the possibility to “escape” antibiotics and host immune and are responsible for the majority of hospital or public infections (Rice, 2008; Da Rosa et al., 2023). Among these “ESKAPE” pathogens, *P. aeruginosa* was considered a “critical” pathogen in the bacterial pathogens list of the World Health Organization (WHO), which emphasized the emergency for researching and developing novel antibacterial agents to cure *P. aeruginosa* infection (Tacconelli et al., 2018). The quorum sensing (QS) system of *P. aeruginosa* regulates the production of virulence factors and biofilm formation to adapt to diverse habitats causing chronic diseases and antibiotic resistance (Morita et al., 2014; Qin et al., 2014). QS is a bacterial cell-to-cell communication that is an extensive signaling in which diffusible signaling molecules are secreted into the environment and sensed until the density reaches the threshold by a homologous cellular receptor (Waters and Bassler, 2005; Asfahl et al., 2017). After binding to these signaling molecules, the receptor complex activates the transcription of target genes involved in processes of secreting virulence factors, biofilm formation, and drug resistance (Papenfort and Bassler, 2016; Smith and Schuster, 2022). Therefore, QS inhibition has already been considered a potent strategy for avoiding drug resistance to combat *P. aeruginosa* infections (Ji et al., 2023). Quorum sensing inhibitors (QSIs) can attenuate the production of virulence factors and inhibit biofilm formation of pathogens without affecting their growth, leading to avoidance of multidrug resistance, which has attracted extensive attention. Aminoglycosides and β-lactam agents are usually the first line to treat *P. aeruginosa* infections (Foulkes et al., 2022; Tamma et al., 2022). Combined with aminoglycoside antibiotics, hordenine as a typical QSI could downregulate genes involved in QS and biofilm formation to enhance the susceptibility of aminoglycosides against *P. aeruginosa* PAO1 biofilm formation (Zhou et al., 2018b). Therefore, the combination of the aminoglycoside antibiotics with a QSI may be more effective than antibiotics alone in curing infections caused by *P. aeruginosa*.

Natural products are one of the essential sources of QSIs. Endophytic fungi are an under-explored source of bioactive natural products, offering opportunities for the discovery of novel QSIs (Joo et al., 2021; Pellissier et al., 2021). Rajesh and Ravishankar Rai reported that the endophytic fungi *Fusarium graminearum* and *Lasiodiplodia* sp. isolated from *Ventilago madraspatana* Gaertn present anti-QS activity (Rajesh and Ravishankar Rai, 2013). 1-(4-Amino-2-hydroxyphenyl)ethanone isolated from the endophytic fungus *Phomopsis liquidambari* presents QSI activity against *P. aeruginosa* (Zhou et al., 2021). In addition, Koh et al. reported that *Areca catechu* L. fruit extracts inhibit QS in both *Chromobacterium violaceum* CV026 and *P. aeruginosa* PAO1 (Koh and Tham, 2011). Endophytes generate the same various metabolites including QSI with symbiotic plants (Meena et al., 2020). Actinomycin D isolated from an endophyte of *Streptomyces cyaneochromogenes* RC1 isolated from *A. catechu* L. is a novel *P. aeruginosa* QSI (Zeng et al., 2022). However, the QSIs in *A. catechu* L. endophytic fungus are not clear.

Previous reports suggested that the biofilm eradication of the combination of antibiotics and QSIs is better than that of antibiotics alone (Chanda et al., 2017; Zhou et al., 2018b). Resveratrol as a QSI can combine with aminoglycoside to strengthen the bactericidal effect of antibiotics on *P. aeruginosa* (Zhou et al., 2018a). Therefore, the present study aimed to find new QSI form *A. catechu* L. endophytic fungi, which are involved in the inhibition of QS-related virulence factors and biofilm formation of *P. aeruginosa* PAO1. The inhibition of biofilm formation and the eradication of mature biofilm treated with QSI combined with gentamycin or piperacillin were also investigated to increase the antibiotic sensitivity and decrease the pathogenicity of *P. aeruginosa*.

## Materials and methods

### Bacterial strains and culture condition

*C. violaceum* CV026 and *P. aeruginosa* PAO1 were kindly acquired from Q.H. Gong (Ocean University of China). *C. violaceum* CV026 was cultured in Luria–Bertani (LB) broth at 28°C, 180 rpm, for 17 h to prepare overnight cultures. *P. aeruginosa* PAO1 was incubated in LB broth at 37°C, 180 rpm, for 20 h to prepare overnight cultures.

### Isolation and extraction of endophytic fungus

The surface of the *A. catechu* L. fruits was disinfected with 75% (v/v) ethanol for 1 min, then soaked in 0.2% mercuric chloride solution for 10 min, and washed three times with sterilized water to remove the residual mercuric chloride (Li et al., 2016). The surface-sterilized samples were transferred onto a dried sterile filter paper to remove the liquid on the surface. After that, the fruits were cut into small lumps (approximately 1.0 × 1.0 cm). Subsequently, each lump was carefully settled on a potato dextrose agar (PDA) plate separately and incubated at 28°C for 15 d. During the incubation period, the samples were observed every day, and every newly emerged fungal spot was immediately picked out and carefully transferred to another fresh PDA plate. After incubation, the resulting fungal strains were further purified and then maintained at 4°C (**Supplementary Figure S1**). Sterile conditions were needed during the process of all operations.

The isolated strains were cultured in 200 mL of Fungus NO.2 medium at 180 rpm, 28°C, for 1 week (Zhou et al., 2021). The cultures were centrifuged at 8,000 rpm, 4°C, for 10 min. The supernatant was added to an equal volume of ethyl acetate to be extracted three times, and the pooled extraction solvent was removed by rotary evaporation to collect the crude extracts. The crude extracts were dissolved in 1 mL methanol and filtered through a 0.22-µm nylon filter for the next QSI screening assay.

### Screening and identification of the QSI active strains

*C. violaceum* CV026 was used as a sensor to test the QSI activities (Zeng et al., 2022). The overnight culture of *C. violaceum* CV026 was adjusted to OD_620_ = 0.1, then 1 mL culture was added to 100 mL LB agar medium with exogenous signaling molecule *N*-hexanoyl-l-homoserine lactone (final concentration 5 μM) and mixed thoroughly evenly, and the mixture was poured into Petri dishes. The crude extracts (20 μL) were added to each Oxford cup. Equal volume methanol was used as the negative control. After incubating at 28°C for 24 h, the pigment inhibition zone of *C. violaceum* CV026 was observed and recorded (Zhou et al., 2021).

The endophytic fungus was cultured on a PDA plate before DNA extraction. The total DNA was extracted and amplified by PCR with primers ITS1 (5′-TCCGTAGGTGAACCTGCGG-3′) and ITS4 (5′-TCCTCCGCTTATTGATATGC-3′) for analyzing the internal transcribed spacer (ITS) region of the nuclear ribosomal DNA (Zhang et al., 2016). The PCR product of endophytic fungus was sequenced by Tsingke Biotechnology Co., Ltd. (Beijing, China). The obtained sequences were aligned using MEGA-X to construct a phylogenetic tree.

### Isolation and identification of the QSI active compound

The endophytic fungus was cultured in rice solid medium for 15 d. After that, the metabolites of endophytic fungus were extracted with ethyl acetate and then purified by normal and reversed-phase C18 silica gel chromatography, high-performance liquid chromatography (HPLC), and Sephadex LH-20 with QS inhibiting bio-guided assays. The purified active compound was identified by liquid chromatography–mass spectrometry (LC-MS) and NMR and numbered PT22. PT22 was dissolved in dimethyl sulfoxide (DMSO) for 100 mg/mL.

### Minimum inhibitory concentrations and growth curves measurement

The minimum inhibitory concentrations (MICs) of PT22 and antibiotics against *P. aeruginosa* PAO1 were performed according to the Clinical and Laboratory Standards Institute (CLSI 2023) method. Different final concentrations of PT22 and antibiotics (gentamycin and piperacillin) were twofold serially diluted in Müller–Hinton (MH) medium mixed with bacterial overnight cultures (0.1% volume percent, OD_620_ = 0.5). The mixtures were transferred to 96-well plates and incubated at 37°C, 180 rpm, for 24 h. DMSO served as a negative control. After incubation, the value of OD_620_ of each well was determined at each dose. The sub-MICs were considered for further assays.

The growth curves measurement assays were performed according to the reported methods with some modifications (Dahibhate et al., 2022). Overnight cultures of *P. aeruginosa* PAO1 were inoculated into 5 mL fresh LB broth with 0.1% volume percent and supplemented with different concentrations of PT22 (0.50 mg/mL, 0.75 mg/mL, 1.00 mg/mL, and 2.00 mg/mL). Hordenine (1.00 mg/mL) and DMSO served as positive and negative controls, respectively (Zhou et al., 2018b). The mixtures were transferred to 96-well plates and incubated at 37°C, 180 rpm, for 28 h. For every hour, the absorbance at OD_620_ was assessed by a microplate reader (Biotek EPOCH2, Winooski, VT, USA).

### Signaling molecule level assay

The effects of PT22 on the synthesis of *N*-butanoyl-l-homoserine lactone (C4-HSL) and *N*-(3-oxododecanoyl)-l-homoserine lactone (3-oxo-C12-HSL) in *P. aeruginosa* PAO1 were detected by a high-performance liquid chromatography–mass spectrometry system (LCMS-IT-TOF, SHIMADZU, Kyoto, Japan) equipped with a C18 column (100 × 2.1 mm, particle 5 μm). The relative levels of C4-HSL and 3-oxo-C12-HSL were identified and determined according to the retention time (Rt) of standards and their MS/MS^2^ fragment ions. Overnight culture of 100 µL was added to 100 mL of LB broth containing PT22 (0.50 mg/mL, 0.75 mg/mL, and 1.00 mg/mL) and inoculated at 37°C, 180 rpm, for 20 h. DMSO served as the negative control. After that, the fermentation broths were centrifuged at 10,000 rpm at 4°C for 10 min. The supernatant was mixed with an equal volume of acidified ethyl acetate (0.5% formic acid) three times. The resulting solvents were dried, then dissolved in methanol, and filtered through a 0.45-µm nylon filter (Zhou et al., 2018a). Mobile phase A was double distilled water (0.1% formic acid), and mobile phase B was methanol. The injection volume was 10 µL. The flow rate was 0.8 mL/min. The gradient for standard C4-HSL was set as follows: 1–10 min, 10% B; 15–20 min, 10%–30% B. The gradient for authentic 3-oxo-C12-HSL was set as follows: 1–10 min, 20% B; 15–20 min, 20%–40% B. The results were normalized to the relative quantification of negative control.

### Inhibition of virulence factor assay

The overnight cultures of *P. aeruginosa* PAO1 (0.50 mL) were inoculated 0.1% into 50 mL LB broth supplemented with PT22, hordenine, and DMSO (Zhou et al., 2023a), and their final concentrations of PT22 were 0.50 mg/mL, 0.75 mg/mL, and 1.00 mg/mL, respectively. The reported QSI hordenine (1.00 mg/mL) and DMSO served as positive and negative controls, respectively. After incubation at 37°C, 180 rpm, for 20 h, the cultures were centrifuged at 4°C, 10,000 rpm, for 10 min and filtered through a sterilized 0.22-μm nylon syringe filter. The cell-free supernatant was collected for virulence factor assays.

The measurement of pyocyanin was performed according to reports with some modifications (Ahmed et al., 2019). Chloroform (1.2 mL) was added to 2 mL supernatant and vortexed until the color of the chloroform phase was changed. After centrifugation at 10,000 rpm, 1 mL chloroform phase was then transferred to a new tube and added equal volume of 0.2 M of hydrochloric acid (HCl). The tubes were vortexed until the color of the chloroform phase was changed. The mixtures were centrifuged at 4°C, 10,000 rpm, for 10 min. The absorbance of the supernatant was determined at OD_520_ using a microplate reader (Biotek EPOCH2, USA).

Alginate production was estimated according to the method with slight modifications (Gopu et al., 2015). In brief, 100 µL supernatant was added to 600 µL of boric acid–sulfuric acid mixing solution (4:1, V/V). The mixture was added to 20 µL of 0.2% of carbazole suspended in ethanol, vortexed for 30 s, and then incubated at 55°C for 30 min. The absorbance of the mixture was measured at OD_530_ (Biotek EPOCH2, USA).

Siderophore quantitative assay was performed as the chrome azurol S method with minor modifications (Yin et al., 2022). The supernatant (2 mL) was mixed with an equal volume of chrome azurol S (CAS) assay solution and maintained for 30 min in darkness. The absorbance of the mixture was measured at OD_630_ with a microplate reader (Biotek EPOCH2, USA).

The inhibitory effects of PT22 on elastase activity were determined based on the experimental protocol given by the elastase ELISA kit (Mlbio, Shanghai, China).

Protease activities were measured via reported methods with some modifications (Zhou et al., 2018a). Briefly, 150 μL supernatant was added to 250 µL 0.3% azocasein dissolved in 0.1 M phosphate-buffered saline (PBS; pH 8.0) and incubated at 37°C for 3 h. To quickly terminate the reaction, 1.2 mL of 10% trichloroacetic acid was added and immersed in ice for 20 min. After centrifugation at 4°C, 10–000 rpm, for 10 min, an equal volume of 1 M sodium hydroxide solution was mixed with the supernatant, and the absorbance at OD_440_ was measured using a microplate reader (Biotek EPOCH2, USA).

Rhamnolipid contents were measured using reported methods with some modifications (Zhou et al., 2018a). HCl at a concentration of 6 M was added to the supernatant (5 mL) to pH 2.0. The mixtures were maintained at 4°C for 15 h and then centrifuged at 10,000 rpm, 4°C, for 20 min. The supernatant was removed, and the precipitate was dissolved in 1 mL of methanol. After the addition of 4 mL of sulfuric acid anthrone solution (0.2% anthrone dissolved in 85% sulfuric acid), the solution was maintained in a boiling water bath for 15 min, and the absorbance at OD_625_ was measured using a microplate reader (Biotek EPOCH2, USA).

### Motility assays

The motility assays were evaluated according to the reports (Chadha et al., 2022). Overnight cultures (2 μL) of *P. aeruginosa* PAO1 were inoculated into the center of the swimming agar (1% tryptone, 0.5% NaCl, and 0.3% agar), swarming agar (1% peptone, 0.5% NaCl, 0.5% glucose, and 0.5% agar), and twitching agar (LB broth with 1% agar) in the absence or presence of PT22 (0.50 mg/mL, 0.75 mg/mL, and 1.00 mg/mL). Hordenine (1.00 mg/mL) and DMSO served as positive and negative controls, respectively. The plates were incubated at 37°C for 17 h, and the diameters of the colony were recorded.

### Real−time quantitative PCR analysis

The effects of PT22 on the expression levels of QS-related genes in *P. aeruginosa* PAO1 were investigated with previous descriptions (Zhou et al., 2018a; Zhou et al., 2023b). Briefly, overnight culture was inoculated to 50 mL of LB broth containing PT22 (1.00 mg/mL) and incubated at 37°C, 180 rpm, for 20 h. DMSO served as the negative control. The RNA extraction kit (Sangon Biotech, Shanghai, China) was used to extract total RNA. The cDNA was obtained by Maxima Reverse Transcriptase (Thermo Scientific, Waltham, MA, USA). RT-qPCR was performed using SG Fast qPCR Master Mix Kit (Sangon Biotech, China) on a fluorescent qPCR instrument (StepOnePlus, ABI, Foster City, CA, USA). The gene *rps*L was used as an internal control to normalize RT-qPCR data. The primers used are listed in **Supplementary Table S2**. The relative expression levels of target genes were calculated via the 2^−ΔΔCt^ method.

### Molecular docking

Molecular docking analysis was performed using the AutoDock Vina (Trott and Olson, 2010). The 3D structures of all ligands were drawn using Chem 3D software. The 3D structures of the receptor proteins LasI, LasR, RhlR, and PqsR were downloaded from the Protein Data Bank (ID: 1RO5, 2UV0, 8B4A, and 6Q7U). The 3D structures of RhlI and MexB (ID: P54291 and P52002) were obtained from UniProt (https://www.uniprot.org).

### Microdilution checkerboard assay

The microdilution checkerboard methods were performed to evaluate the effects of antibiotics in combination with PT22 (Chadha et al., 2022). Different final concentrations of PT22 and antibiotics (gentamycin and piperacillin) were twofold serially diluted in MH medium mixed with bacterial overnight cultures. The mixtures were transferred to 96-well plates, and each well that contained antibiotic and PT22 was incubated at 37°C for 24 h. The fractional inhibitory concentration indexes (FICIs) were calculated for each antibiotic in each combination.

### Biofilm inhibition assay

Biofilm inhibition assays were carried out according to the reports with minor modifications (Moreno-Chamba et al., 2023; Zhou et al., 2023a). For biofilm formation inhibition assays, overnight cultures were inoculated into tryptic soy broth (TSB) broth supplemented with PT22 (0.50 mg/mL, 0.75 mg/mL, and 1.00 mg/mL), gentamycin (4.00 μg/mL), piperacillin (4.00 μg/mL), the combination of 4.00 μg/mL gentamycin and 0.50 mg/mL PT22 (gentamycin + PT22), and the combination of 4.00 μg/mL piperacillin and 0.50 mg/mL PT22 (piperacillin + PT22). Hordenine (1.00 mg/mL) and DMSO served as positive and negative controls, respectively. The mixtures were transferred to the 96-well plates (150 μL), and after incubation at 37°C for 24 h, the excess TSB medium was removed. The biofilms were washed with sterile PBS and stained with 0.05% (w/v) crystal violet for 15 min. Then, the biofilms were washed with PBS to remove excess crystal violet. After being dried at 60°C, 200 µL of 95% ethanol was used to dissolve crystal violet. The absorbance was determined at OD_570_ using a microplate reader (Biotek EPOCH2, USA).

For biofilm eradication assays, overnight cultures were inoculated to fresh TSB broth, and 150 µL was transferred to 96-well plates followed by incubation at 37°C for 24 h. After that, the suspension cultures were removed, and the residual biofilms were gently washed with sterile PBS. Then, 150 μL fresh TSB broth was added to well containing PT22 (0.50 mg/mL), gentamycin (4.00 μg/mL), piperacillin (4.00 μg/mL), gentamycin (4.00 μg/mL) + PT22 (0.50 mg/mL), and piperacillin (4.00 μg/mL) + PT22 (0.50 mg/mL). The plates were incubated at 37°C for 24 h. After incubation, the biofilm mass was determined according to the biofilm formation inhibition assays.

### Microscopic analysis

Scanning electron microscopy (SEM) and confocal laser scanning microscopy (CLSM) were used to investigate and visualize biofilms following exposure to drugs (Chadha et al., 2023). Briefly, overnight cultures were inoculated to LB broth supplemented with PT22 (0.50 mg/mL, 0.75 mg/mL, and 1.00 mg/mL), gentamycin (4.00 μg/mL), piperacillin (4.00 μg/mL), gentamycin (4.00 μg/mL) + PT22 (0.50 mg/mL), and piperacillin (4.00 μg/mL) + PT22 (0.50 mg/mL). DMSO served as a negative control. The mixtures were transferred to the 24-well plates with circular coverslips and incubated at 37°C for 24 h.

For SEM assay, after incubation, the mature biofilms on the coverslips were gently washed with PBS to remove the planktonic bacteria; 2.5% glutaraldehyde was used to fix the mature biofilms for 12 h and then removed followed by gentle washing with PBS (pH 7.2). The resulting biofilms were sequentially dehydrated using a gradient of ethanol (50%, 70%, 90%, and 100%). The coverslips were freeze-dried, gold-coated, and visualized under SEM (S4800, HITACHI, Tokyo, Japan).

For CLSM, the mature biofilms on the coverslips were stained with 0.1 mg/mL of acridine orange solution and ethidium bromide solution (V/V = 1:1) for 15 min in darkness, and the images were observed by CLSM (TCS SP8, Leica, Wetzlar, Germany).

### Cytotoxicity assay

Cytotoxicity was measured via CCK-8 assay using RAW 264.7 cells incubated in Dulbecco’s Modified Eagle’s Medium (DMEM) according to the reported method, with some modifications (Fong et al., 2019). Briefly, RAW 264.7 cells were grown in DMEM and supplemented with 10% fetal bovine serum (Gibco, Grand Island, NY, USA) at 37°C and 5% CO_2_. The cells were transferred into 96-well microplates with 1 × 10^5^ cells/well. After being incubated for 24 h, the cells were washed with PBS and treated with PT22 (0.50 mg/mL, 0.75 mg/mL, and 1.00 mg/mL) in 100 µL DMEM. DMSO was used as the negative control. After continuous incubation at 37°C and 5% CO_2_ for 24 h, 10 μL of CCK-8 solution was added to each well, followed by incubation at 37°C for an additional 1 h for cell viability measurement. Subsequently, solution absorbance was measured using a microplate reader (Biotek EPOCH2, USA) at 450 nm.

### *Galleria mellonella* survival assay

*G. mellonella* larvae have been considered a traditional model to investigate microbial infections *in vivo* (Tsai et al., 2016). The survival rates of the larvae after the treatment of PT22 (0.50 mg/mL, 0.75 mg/mL, and 1.00 mg/mL), antibiotics (4.00 μg/mL gentamycin and 4.00 μg/mL piperacillin) alone, gentamycin (4.00 μg/mL) + PT22 (0.50 mg/mL), and piperacillin (4.00 μg/mL) + PT22 (0.50 mg/mL) were used to evaluate the effects on *P. aeruginosa* PAO1 infection according to the reports with some modifications (Ménard et al., 2021). DMSO served as a negative control. Briefly, overnight cultures of *P. aeruginosa* PAO1 were centrifuged at 4°C, 10,000 rpm, for 10 min, resuspended in sterile 0.95% NaCl solution, and adjusted to 10^6^ CFU/mL. After being disinfected externally with 75% ethanol, a 10-μL aliquot was injected into larvae using a Hamilton syringe, and a 10-μL dose of a single drug type or drug combination was injected directly into larvae at 1 h post-infection. Mortality was monitored each 24 h for 120 h. The controls were injected with 10 µL of sterile 0.95% NaCl solution.

### Statistical analysis

All experiments were performed in biological triplicates, and data were presented as means ± standard deviation (SD). Statistical differences were evaluated with a one-way analysis of variance ANOVA test or *t*-test using GraphPad Prism 8 (GraphPad Software Inc., La Jolla, CA, USA). *p* ≤ 0.05 was considered to be statistically significant (*p ≤ 0.05, **p ≤ 0.01, ***p ≤ 0.001, and ****p ≤ 0.0001).

## Results

### Identification of the QSI active strain

**Figure 1:**
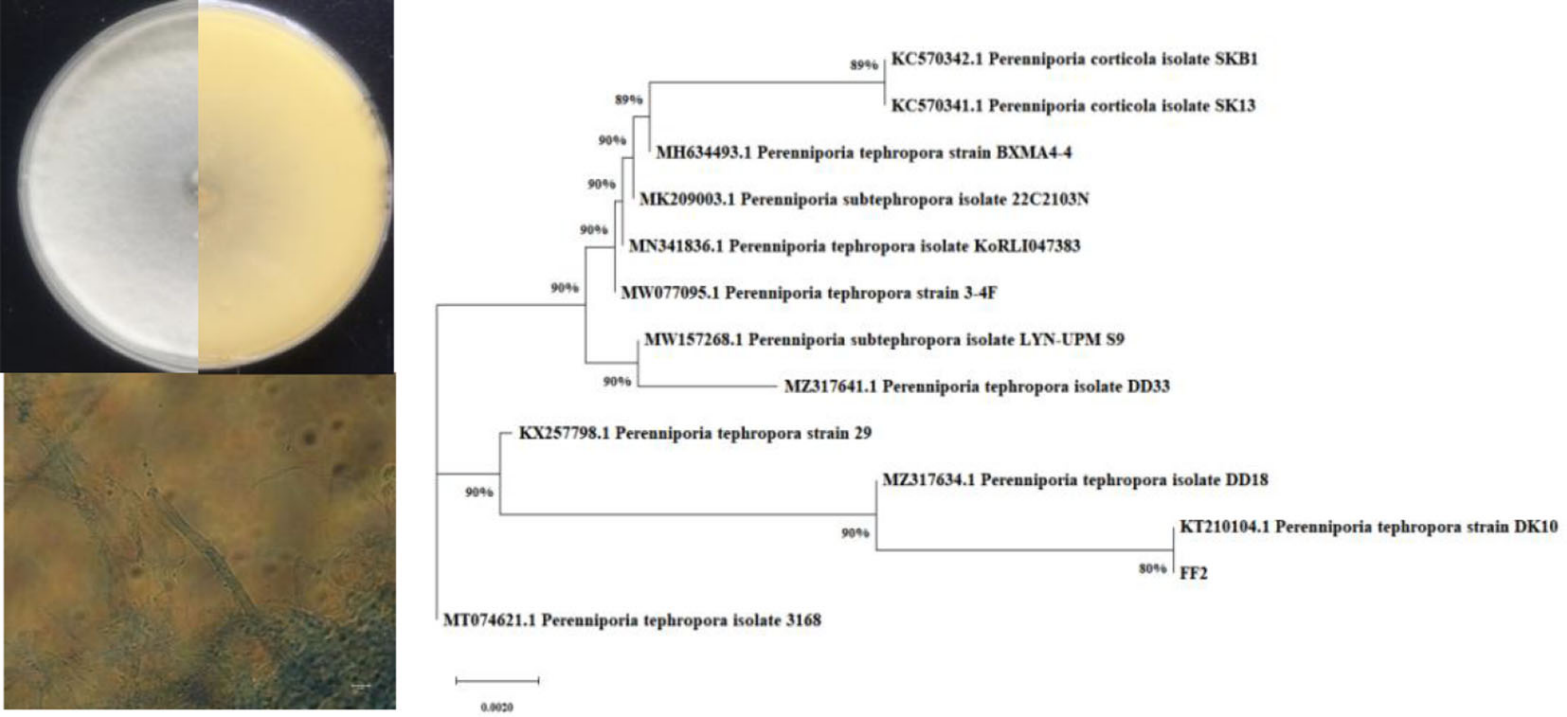
The phylogenetic tree and morphological features of *Perenniporia tephropora* FF2.

The active endophytic fungus FF2 was isolated from the fruits of *A. catechu* L. Morphologically, a colony of strain FF2 on the PDA plate is radial with white substrate mycelia and has a leathery texture (**Figure 1**). The phylogenetic tree was constructed (**Figure 1**) based on the ITS rRNA gene sequencing and the blast results. The strain FF2 was identified as *Perenniporia tephropora* (NCBI accession number: OR349622).

### Purification and identification of QSI active compound

A total of 51.1 mg of QSI active compound PT22 was obtained under bio-guided screenings (**Supplementary Figure S2**), it was a yellow powder, and its high-resolution electrospray ionization mass spectrometry (HR-ESI-MS) was 154.0160 [M]^−^ (calcd for C_6_H_5_NO_4_, 155.1082). By comparing the data from HR-ESI-MS and NMR spectra (**Supplementary Figure S3**) with those of previous studies (Koálová et al., 1992), the chemical was identified as 1H-pyrrole-2,5-dicarboxylic acid (**Supplementary Figure S3**).

### Determination of MICs and growth curves

**Figure 2:**
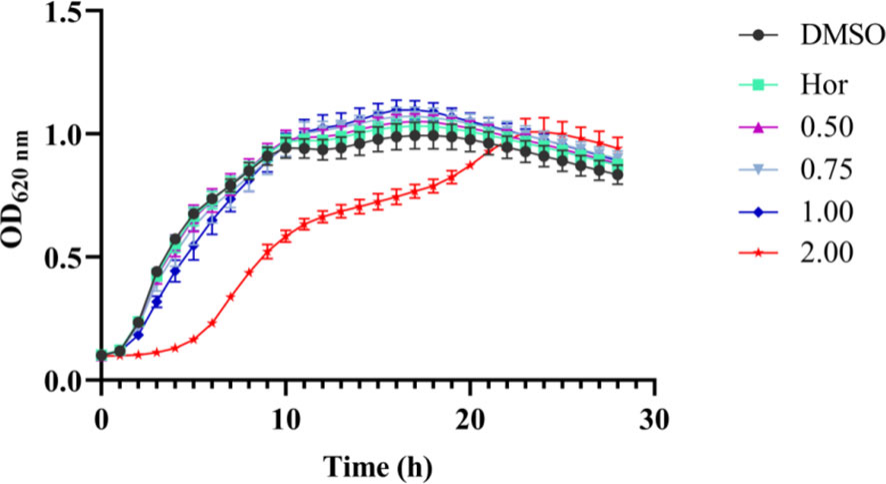
Effects of PT22 (0.50 to 2.00 mg/mL) on the growth curves of *Pseudomonas aeruginosa* PAO1. Hordenine (Hor; 1.00 mg/mL) and DMSO served as positive and negative controls, respectively. DMSO, dimethyl sulfoxide.7.

The MIC of PT22 against *P. aeruginosa* PAO1 was more than 2.00 mg/mL, and hence, the concentrations for studying the QSI experiments should be sub-MIC. The results of the growth curves showed that PT22 presented no effect on *P. aeruginosa* PAO1 growth from 0.50 to 1.00 mg/mL (**Figure 2**). Therefore, the concentrations of 0.50 mg/mL, 0.75 mg/mL, and 1.00 mg/mL were selected for the QSI active evaluation. In addition, the MICs of gentamycin and piperacillin were 8.00 μg/mL (**Supplementary Table S1**).

### Inhibition of signaling molecule secretion

**Figure 3:**
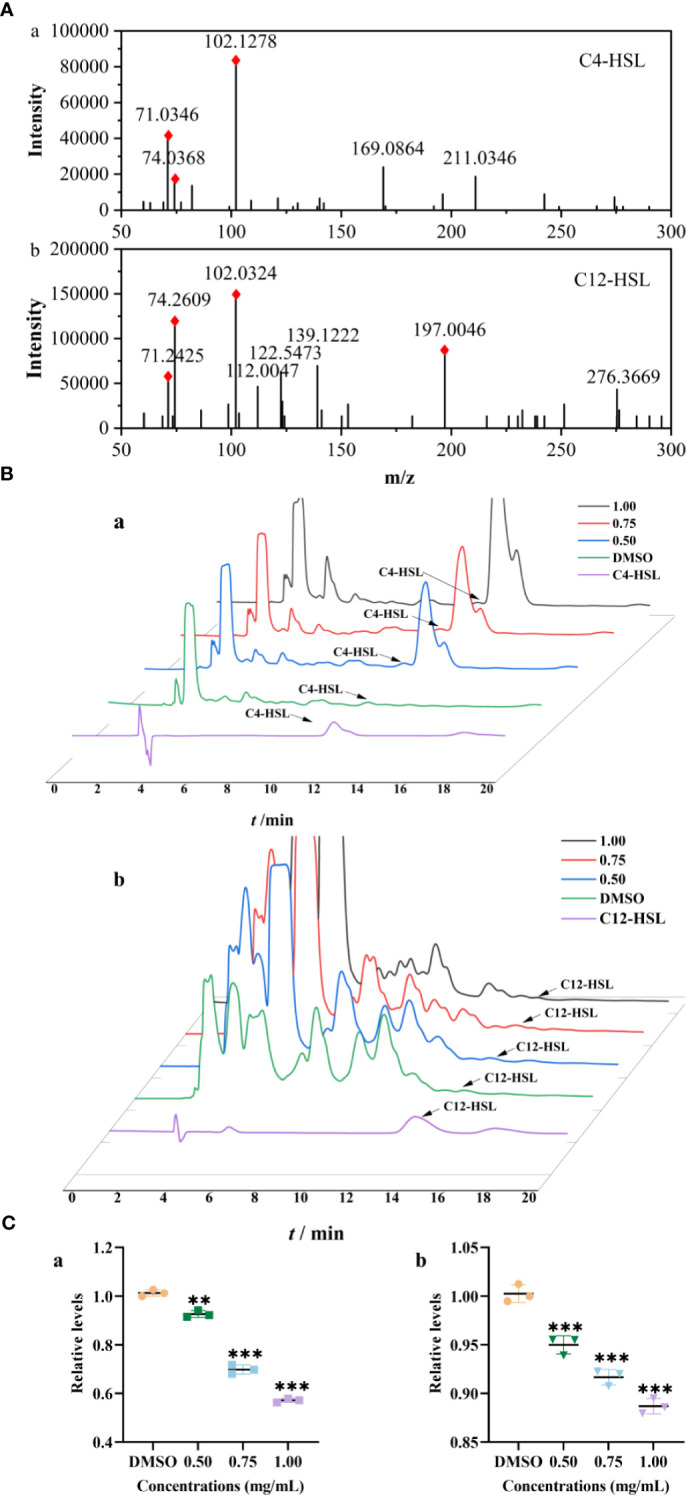
Relative quantification of C4-HSL and 3-oxo-C12-HSL by HPLC-MS/MS^2^. Product ion spectra of C4-HSL (a) and 3-oxo-C12-HSL (b) **(A)**. The chromatograms of AHLs produced by *Pseudomonas aeruginosa* PAO1 treated with PT22 (0.50 mg/mL, 0.75 mg/mL, and 1.00 mg/mL) **(B)**. Quantitative analysis of C4-HSL (a) and 3-oxo-C12-HSL (b) exposed to PT22 (0.50 mg/mL, 0.75 mg/mL, and 1.00 mg/mL) **(C)**. DMSO served as negative control. **p < 0.01 and ***p < 0.001 compared to the DMSO group by one-way ANOVA. HPLC, high-performance liquid chromatography; AHLs, acylated homoserine lactones; DMSO, dimethyl sulfoxide.

The Rts of C4-HSL and 3-oxo-C12-HSL in strain were 11.99 and 13.56 min, respectively, which were identified with standards and their MS/MS^2^ fragment ions (**Figures 3A, B**). Compared to those of the control group, the relative levels of C4-HSL treated with PT22 (0.50 mg/mL, 0.75 mg/mL, and 1.00 mg/mL) were reduced by 8.59%, 31.41%, and 44.12%, respectively (**Figure 3C**, a). Correspondingly, the levels of 3-oxo-C12-HSL were reduced by 11.55% after PT22 treatment at 1.00 mg/mL (**Figure 3C**, b). This demonstrates the PT22 may impede C4-HSL production, resulting in QSI activity.

### Inhibition of virulence factors

**Figure 4:**
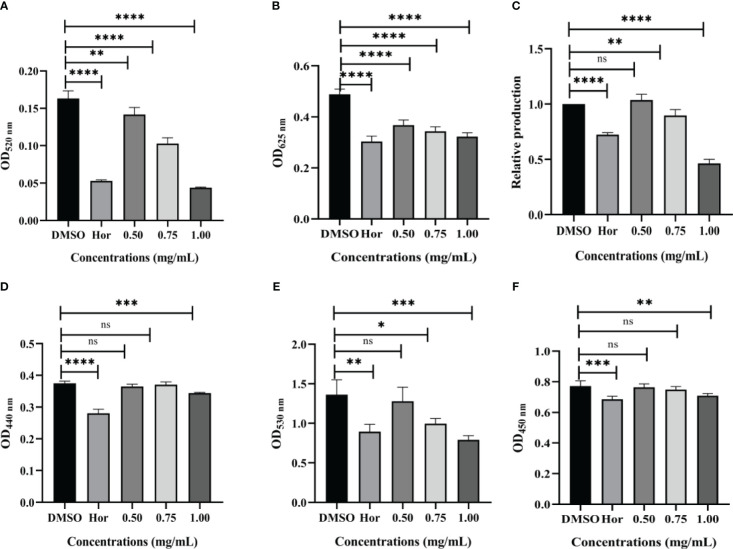
Effects of PT22 (0.50 mg/mL, 0.75 mg/mL, and 1.00 mg/mL) on virulence factors of *Pseudomonas aeruginosa* PAO1, including pyocyanin **(A)**, rhamnolipid **(B)**, siderophores **(C)**, elastase **(D)**, alginate **(E)**, and protease **(F)**. Hordenine (b) and DMSO (a) served as positive and negative controls, respectively. *p < 0.05, **p < 0.01, ***p < 0.001, and ****p < 0.0001 compared to the DMSO group by one-way ANOVA. DMSO, dimethyl sulfoxide. “ns” means no significance.

The virulence factors are the key element to help bacteria infect, colonize, and survive. There were significant reductions in virulence factors of *P. aeruginosa* PAO1 after being exposed to PT22. As shown in **Figure 4A**, the pyocyanin contents were dramatically reduced by 13.17%, 37.06%, and 73.05% after treatment with different concentrations (0.50 mg/mL, 0.75 mg/mL, and 1.00 mg/mL, respectively) of PT22, whereas hordenine presented inhibition by 67.54% at 1.00 mg/mL. Hordenine (1.00 mg/mL) treatment led to the reduction of the rhamnolipid level by 37.95%. Additionally, rhamnolipid production declined by 24.75%, 29.66%, and 34.06% at concentrations of 0.50 mg/mL, 0.75 mg/mL, and 1.00 mg/mL, respectively (**Figure 4B**). The relative levels of the siderophores were decreased by 10.35% and 53.59% at 0.75 and 1.00 mg/mL of PT22, respectively, while the positive control hordenine (1.00 mg/mL) showed the depletion in siderophores by 27.7% (**Figure 4C**). The protease and elastase activities were reduced by 8.27% and 8.16%, respectively, after being treated with PT22 at 1.00 mg/mL (**Figures 4D, F**), whereas hordenine (1.00 mg/mL) presented inhibition by 25.15% and 11.17%, respectively. Approximately 26.98% and 42.02% reductions of alginate were observed with exposure of 0.75 and 1.00 mg/mL of PT22, respectively (**Figure 4E**). Moreover, the hordenine (1.00 mg/mL) presented a depletion of alginate production by 34.46%. Further, with the concentration increasing, the inhibitory effects of PT22 on virulence factors except protease and elastase of *P. aeruginosa* PAO1 were gradually increased in dose-dependent manners.

### Inhibition of motility

**Figure 5:**
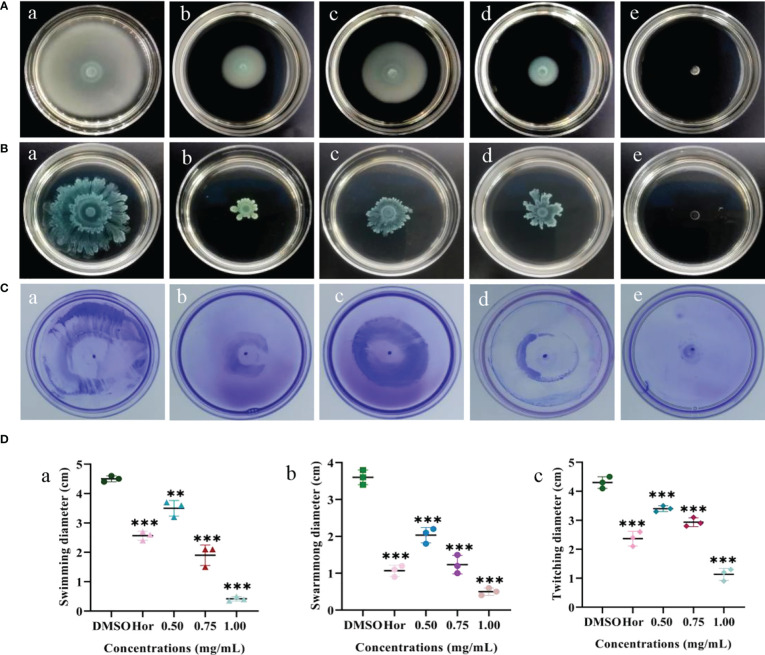
Effects of PT22 [0.50 (c), 0.75 (d), and 1.00 (e) mg/mL) on swimming **(A)**, swarming **(B)**, and twitching **(C)** of *Pseudomonas aeruginosa* PAO1. Hordenine (b) and DMSO (a) served as positive and negative controls, respectively. The diameters of the swimming (a), swarming (b), and twitching (c) zones were recorded **(D)**. **p < 0.01 and ***p < 0.001 compared to the DMSO group by one-way ANOVA. DMSO, dimethyl sulfoxide.

Flagella are able to help planktonic bacteria colonize on surfaces. The effects of PT22 on swimming, swarming, and twitching of *P. aeruginosa* PAO1 were evaluated (**Figures 5A–C**). There was a significant depletion in swimming motility of 22.22%, 57.78%, and 90.73% when exposed to PT22 (0.50 mg/mL, 0.75 mg/mL, and 1.00 mg/mL, respectively) (**Figure 5D**, a). The swimming motility of *P. aeruginosa* PAO1 was markedly reduced by 42.96% when exposed to hordenine (1.00 mg/mL). The swarming diameter was decreased by 43.53%, 65.75%, and 86.11% (**Figure 5D**, b). Moreover, when treated with hordenine (1.00 mg/mL), the colony diameter was reduced by 70.36%. For the twitching study, the colony diameter was decreased by 20.93, 31.79, and 73.65% (**Figure 5D**, c). Furthermore, treatment with hordenine (1.00 mg/mL) demonstrated a significant reduction in the colony diameter of 44.95%. There is a significant reduction in motility activity of *P. aeruginosa* PAO1 in response to PT22 in comparison with negative control.

### Effects on gene expression

**Figure 6:**
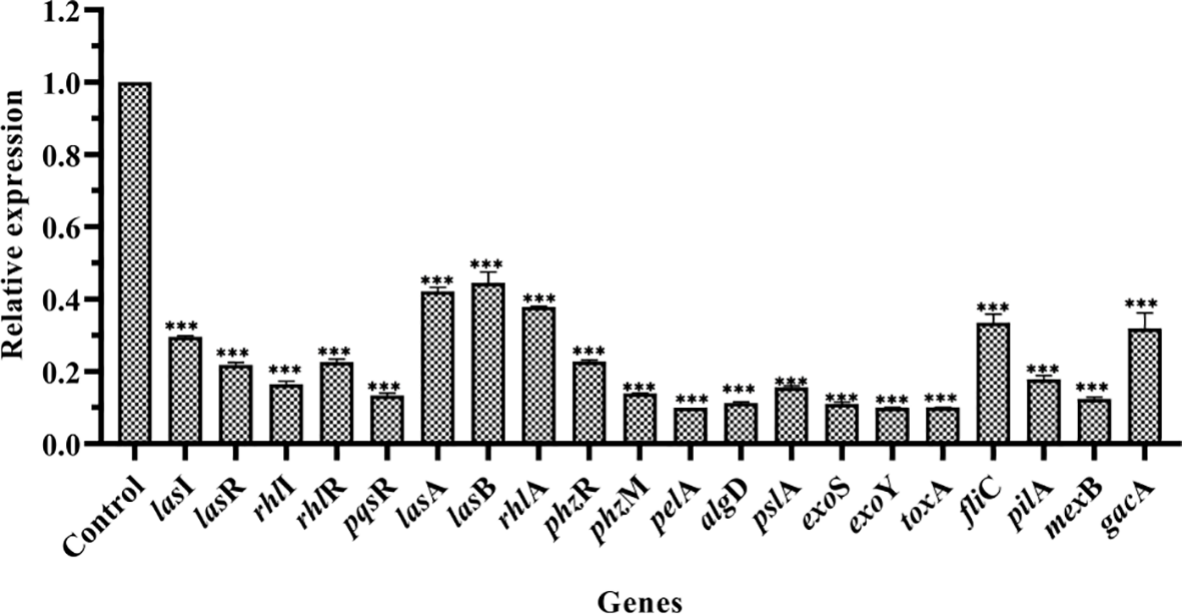
Effects of PT22 1.00 mg/mL on the expression of genes in *Pseudomonas aeruginosa* PAO1. The expression level was versus DMSO control group. The relative expression levels of genes were normalized by the 2^−ΔΔCt^ method. *rps*L was used as an internal reference gene to normalize. The relative expression levels of *las*I, *las*R, *rhl*I, *rhl*R, *pqs*R, *las*A, *las*B, *rhl*A, *phz*R, *phz*M, *pel*A, *alg*D, *psl*A, *exo*S, *exo*Y, *tox*A, *fli*C, *pil*A, *mex*B, and *gac*A were significantly reduced by 70.41%, 78.18%, 83.56%, 77.39%, 86.67%, 57.79%, 55.49%, 62.16%, 77.21%, 86.08%, 90.00%, 88.71%, 84.41%, 88.99%, 89.99%, 89.97%, 66.58%, 82.15%, 87.63%, and 68.14%, respectively. Values are shown as the mean ± SD. ***p < 0.001 compared to the DMSO group by one-way ANOVA. DMSO, dimethyl sulfoxide.

RT-qPCR was performed to study the effect of PT22 on the transcriptional level of QS-related genes in *P. aeruginosa* PAO1 (**Figure 6**). After being exposed to PT22 (1.00 mg/mL) for 20 h, the expression of genes encoding the QS systems (*las*I, *las*R, *rhl*I, *rhl*R, and *pqs*R) was downregulated extremely by 70.41%, 78.18%, 83.56%, 77.39%, and 86.67%, respectively. QS-related genes included protease (*las*A), elastase (*las*B), rhamnolipid (*rhl*A), exotoxin A (*tox*A), and pyocyanin production (*phz*M), which presented significant downregulation by 57.79%, 55.49%, 62.16%, 89.97%, and 86.08%, respectively. Exopolysaccharides produced by *P. aeruginosa* PAO1 mainly including alginate, Pel, and Psl are responsible for biofilm formation. The genes encoding the alginates (*alg*D), Pel (*pel*A), and Psl (*psl*A) treated with PT22 were reduced by 88.71%, 90.00%, and 84.41%, respectively. The genes regulated to motility activity (*fli*C and *pil*A) were downregulated by 66.58% and 82.15%, respectively. Furthermore, *exo*S and *exo*Y encoding the ExoS and ExoY effectors secreted by type III secretion system (T3SS) were downregulated by 88.99% and 89.99%, respectively. The expression level of *mex*B related to the multidrug resistance efflux pump was downregulated by 87.63%. In addition, the expression level of *gac*A related to the GacS/GacA two-component system (TCS), which is a master regulator of virulence, swarming motility, and biofilm formation, was reduced by 68.14% (Wei et al., 2013). The results indicated that the genes related to QS, T3SS, multidrug resistance efflux pump, and the GacS/GacA TCS were downregulated significantly when exposed to PT22 (1.00 mg/mL) for 20 h.

### Molecular docking analysis

**Figure 7:**
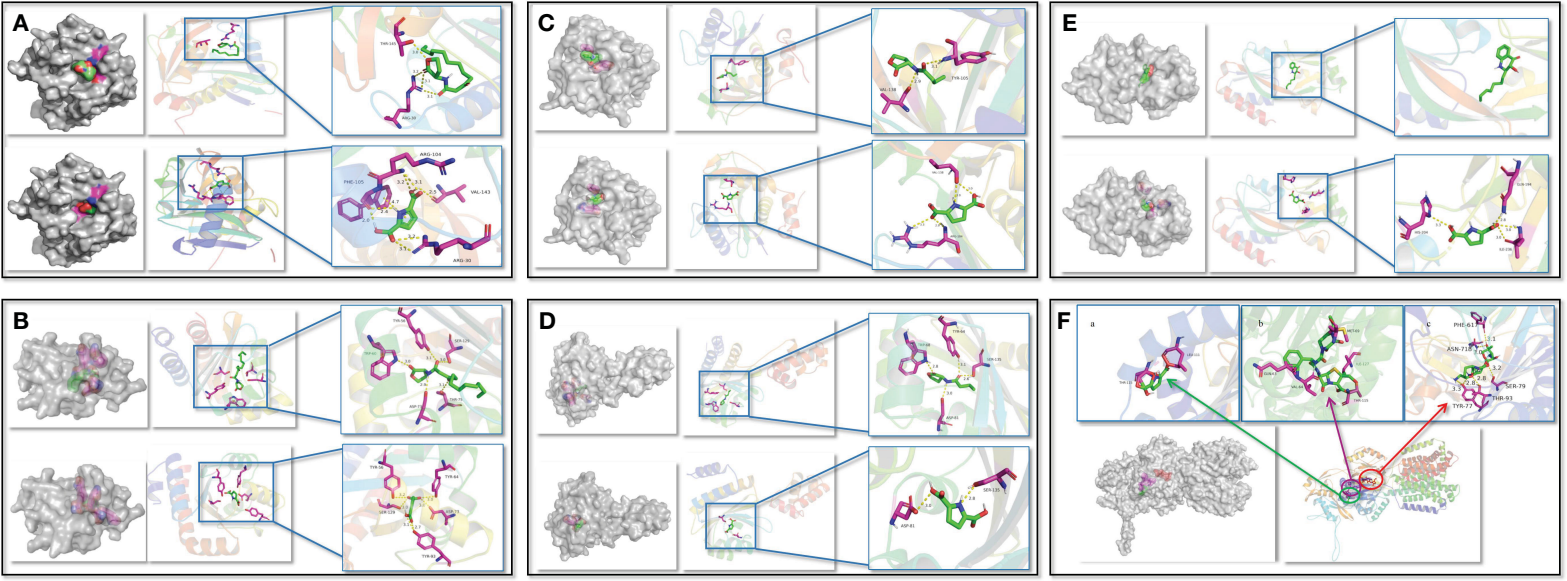
Interactions between QS receptor proteins and various ligands. **(A)** LasI binding to 3-oxo-C12-HSL and PT22. **(B)** LasR binding to 3-oxo-C12-HSL and PT22. **(C)** RhlI binding to C4-HSL and PT22. **(D)** RhlR binding to C4-HSL and PT22. **(E)** PqsR binding to NHQ and PT22. **(F)** MexB binding to PT22 (a), piperacillin (b), and gentamycin (c). The receptor proteins are indicated in gray, and the interaction sites are indicated in multicolor. The hydrogen bonds are shown as yellow dotted lines. The ligands are shown as green sticks, and the interacted residues are shown as magenta sticks. QS, quorum sensing.

To confirm the PT22 against the QS system and multidrug resistance efflux pump of *P. aeruginosa* PAO1, the possibility of PT22 binding interactions with receptor proteins was analyzed by molecular docking. The 3D structures of docking complexes of LasI, LasR, RhlI, RhlR, PqsR, and MexB receptor proteins are shown in **Figure 7**, and the 2D interaction is shown in **Supplementary Figure S4.** The results showed that 3-oxo-C12-HSL binds to the auto-inducer secreting protein LasI via hydrogen bonds at Arg30 and Thr145 residues. PT22 binds to LasI via hydrogen bonds at Arg104, Phe105, Val43, and Arg30 residues (**Figure 7A**). The docking energy between LasI and 3-oxo-C12-HSL is −6.4 kcal/mol, and that between LasI and PT22 is −6.2 kcal/mol (**Supplementary Table S3**). Residues Trp60, Asp73, Thr75, Ser129, and Tyr56 form hydrogen bonds, which are responsible for the docking complex of LasR and 3-oxo-C12-HSL. PT22 binds to the receptor protein LasR via hydrogen bonds at Tyr56, Tyr64, Asp73, Tyr93, and Ser129 residues (**Figure 7B**). The docking energy between LasR and 3-oxo-C12-HSL is −8.6 kcal/mol, which is −7.1 kcal/mol between LasR and PT22 (**Supplementary Table S3**). Hydrogen bonds at Tyr105 and Val138 residues play a major role in C4-HSL binding to RhlI. PT22 binds to RhlI via hydrogen bonds at Val138 and Arg104 residues (**Figure 7C**). The interaction docking energy between RhlI and C4-HSL, and between RhlI and PT22 are −5.4 kcal/mol and −5.1 kcal/mol, respectively (**Supplementary Table S3**). C4-HSL binds to RhlR by hydrogen bonds at Tyr64, Ser135, Asp81, and Trp68 residues. PT22 binds to the receptor protein RhlR via hydrogen bonds at Ser135 and Asp81 residues (**Figure 7D**). The interaction docking energy between RhlR and C4-HSL is −4.4 kcal/mol, and that between RhlR and PT22 is −6.1 kcal/mol. The natural ligand 2-nony-4-quinolone (NHQ) binds to the receptor PqsR via hydrophobic bonds. Residues His204, Ile236, and Gln194 form hydrogen bonds, which are responsible for the docking complex of PqsR and PT22 (**Figure 7E**). The interaction docking energy between PqsR and NHQ, and between PqsR and PT22 is −7.1 kcal/mol and −5.8 kcal/mol, respectively. There are six hydrogen bonds between MexB and gentamycin formed at Phe617, Asn718, Thr93, Tyr77, and Ser79 residues. Piperacillin binds to MexB via covalent bonds of Thr115, Val64, Gln63, Met69, and Ile127. PT22 binds to MexB via covalent bonds at Leu111 and Thr115 residues (**Figure 7F**). The interaction docking energy of gentamycin, piperacillin, and PT22 to MexB was −8.5 kcal/mol, −8.6 kcal/mol, and −5.9 kcal/mol, respectively (**Supplementary Table S4**).

Compared with signaling molecular, PT22 required less docking energy for docking with RhlR but more docking energy for docking with LasR and PqsR, indicating that PT22 can form stable complexes with RhlR as well as exhibit better affinity for RhlR than C4-HSL. There is approximately equal required energy for PT22 and 3-oxo-C12-HSL binding to LasI, and PT22 and C4-HSL binding to RhlI. The lower energy required for the binding of PT22 to LasR compared to RhlR and PqsR suggested that it is easier for PT22 and LasR to form a complex than that of PqsR and RhlR.

### Microdilution checkerboard analysis

The effects of the antibiotics (gentamycin and piperacillin) used in combination with PT22 against *P. aeruginosa* PAO1 were evaluated by FICI values. The FICs of PT22, gentamycin, and piperacillin were 0.50 mg/mL, 4.00, and 4.00 μg/mL, respectively. The FICIs of antibiotics (gentamycin and piperacillin) in combination with PT22 were 0.75, indicating that both gentamycin and piperacillin combined with PT22 have additive interaction against *P. aeruginosa* PAO1 (**Table 1**).

**Table 1:** Fractional inhibitory concentrations (FICs) and FIC indexes (FICIs) for testing synergy between PT22 and antibiotics against *Pseudomonas aeruginosa* PAO1.

Agents	MICs alone (μg/mL)	MICs in combination (μg/mL)	FICs	FICIs	Nature of interaction
Gentamcin	8	4	0.5	0.75	Additivity
Piperacillin	8	4	0.5	0.75	

The FICIs were defined as follows: synergy, FICI ≤ 0.5; additivity, FICI >0.5 to ≤1; no interaction, FICI >1 to ≤4; antagonism, FICI > 4.

### Inhibition and eradication of biofilm

**Figure 8:**
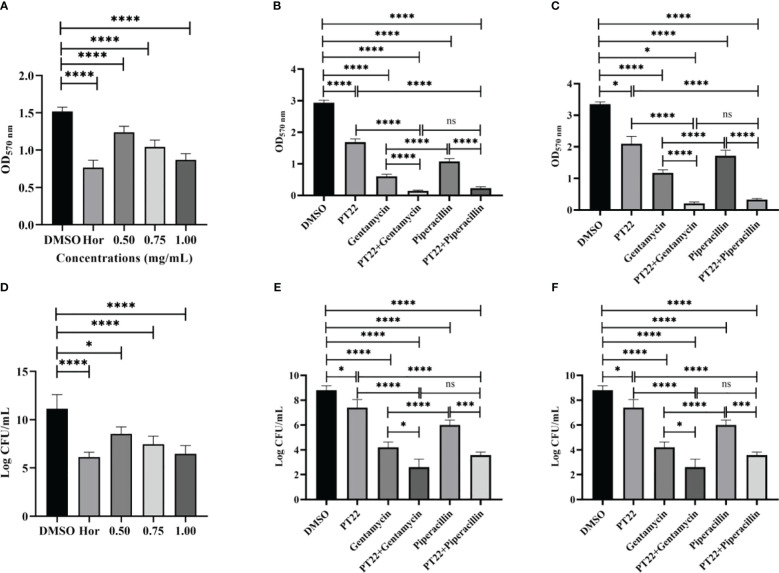
Effects of PT22 (0.50 mg/mL, 0.75 mg/mL, and 1.00 mg/mL) on biofilm formation of *Pseudomonas aeruginosa* PAO1 **(A, B)**. Hordenine and DMSO were served as positive and negative controls, respectively. Additive effects of PT22 (0.5 mg/mL) and gentamycin and piperacillin on inhibition **(C, D)** and eradication **(E, F)** of biofilm of *P. aeruginosa* PAO1. *p < 0.05, ***p < 0.001 and ****p < 0.0001 compared to the DMSO group by one-way ANOVA. DMSO, dimethyl sulfoxide. “ns” means no significance.

The biofilm biomass treated with PT22 (0.50 mg/mL, 0.75 mg/mL, and 1.00 mg/mL) was approximately reduced by 27.89%, 47.59%, and 64.74%, respectively, compared with negative control (**Figure 8A**). Hordenine (1.00 mg/mL) presented significant depletion in biofilms by 75.19%. Furthermore, after being treated with PT22 (0.50 mg/mL, 0.75 mg/mL, and 1.00 mg/mL), the amount of viable cells in the biofilms had a significant reduction compared to the negative control. The inhibition rate was 26.00%, 36.67%, and 46.67% (**Figure 8B**). The amount of viable cells treated with hordenine (1.00 mg/mL) was reduced by 50.00%. The biofilm biomass exposed to gentamycin (4.00 μg/mL) and piperacillin (4.00 μg/mL) was remarkably decreased by 79.47% and 63.24%, respectively. Interestingly, after exposure to gentamycin (4.00 μg/mL) + PT22 (0.50 mg/mL) and piperacillin (4.00 μg/mL) + PT22 (0.50 mg/mL), the biofilm biomass was significantly reduced by 95.06% and 90.16%, respectively, compared with negative control, as well as reduced by 76.00% and 78.46% compared with gentamycin (4.00 μg/mL) and piperacillin (4.00 μg/mL) alone. The biofilm biomass treated with gentamycin (4.00 μg/mL) was significantly reduced compared with piperacillin (4.00 μg/mL). There is no significant difference in inhibiting biofilm formation between treatments of gentamycin (4.00 μg/mL) + PT22 (0.50 mg/mL) and piperacillin (4.00 μg/mL) + PT22 (0.50 mg/mL) (**Figure 8C**). Compared with the control, PT22 (0.50 mg/mL), gentamycin (4.00 μg/mL), piperacillin (4.00 μg/mL), gentamycin (4.00 μg/mL) + PT22 (0.50 mg/mL), and piperacillin (4.00 μg/mL) + PT22 (0.50 mg/mL) treatments resulted in reductions in the amount of viable cells in biofilms by 15.91%, 52.27%, 31.81%, 70.45%, and 59.47%, respectively. After being treated with gentamycin (4.00 μg/mL) + PT22 (0.50 mg/mL) and piperacillin (4.00 μg/mL) + PT22 (0.50 mg/mL), the viable cells in biofilms were significantly reduced by 38.10% and 40.55%, respectively, compared with gentamycin (4.00 μg/mL) and piperacillin (4.00 μg/mL) alone (**Figure 8D**). To assess whether PT22 could enhance mature biofilm sensitivity to antibiotics, the overnight mature biofilms were exposed to PT22 (0.50 mg/mL), gentamycin (4.00 μg/mL), piperacillin (4.00 μg/mL), gentamycin (4.00 μg/mL) + PT22 (0.50 mg/mL), and piperacillin (4.00 μg/mL) + PT22 (0.50 mg/mL) as well as DMSO (negative control) for 24 h. The mature biofilms exposed to gentamycin (4.00 μg/mL) + PT22 (0.50 mg/mL) and piperacillin (4.00 μg/mL) + PT22 (0.50 mg/mL) were dramatically decreased by 93.73% and 90.11% compared with negative control, as well as 82.14% and 80.78% compared with gentamycin (4.00 μg/mL) and piperacillin (4.00 μg/mL) alone, respectively. The mature biofilms treated with gentamycin (4.00 μg/mL) were significantly reduced compared with piperacillin (4.00 μg/mL). There is no significant difference in erasing mature biofilms between those treated with gentamycin (4.00 μg/mL) + PT22 (0.50 mg/mL) and piperacillin (4.00 μg/mL) + PT22 (0.50 mg/mL) (**Figure 8E**). Compared with the control, the amount of viable cells in mature biofilms exposed to gentamycin (4.00 μg/mL) + PT22 (0.50 mg/mL) and piperacillin (4.00 μg/mL) + PT22 (0.50 mg/mL) was reduced by 61.33% and 50.52% and reduced by 31.06% and 40.58% compared with gentamycin (4.00 μg/mL) and piperacillin (4.00 μg/mL) alone, respectively (**Figure 8F**). The results suggested that PT22 enhanced the cytosensitivity of mature biofilm to gentamycin and piperacillin.

**Figure 9:**
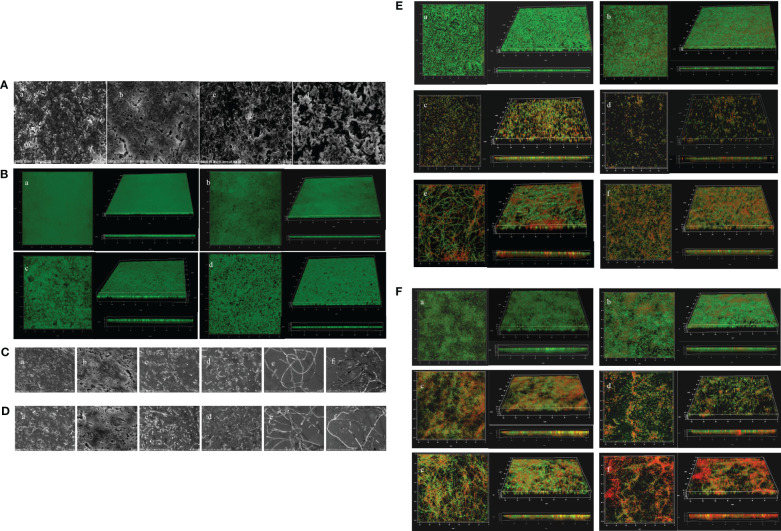
Microscopy images of *Pseudomonas aeruginosa* PAO1 biofilms treated with PT22. SEM images of biofilms treated with DMSO (a), 0.50 (b), 0.75 (c), and 1.00 mg/mL (d) **(A)**. CLSM images of biofilms treated with DMSO (a), 0.50 (b), 0.75 (c), and 1.00 mg/mL (d) **(B)**. Microscopy images of additive effects on biofilm treated with PT22 (0.5 mg/mL) and gentamycin and piperacillin on biofilm formation **(C, E)** and biofilm eradication **(D, F)** of *P. aeruginosa* PAO1. SEM images of biofilm **(C)** treated with DMSO (a), PT22 (0.50 mg/mL) (b), gentamycin (c), PT22 + gentamycin (d), piperacillin (e), and PT22 + piperacillin (f) **(C, D)**. CLSM images of biofilms treated with DMSO (a), PT22 (0.50 mg/mL) (b), gentamycin (c), PT22 + gentamycin (d), piperacillin (e), and PT22 + piperacillin (f) **(E, F)**. DMSO, dimethyl sulfoxide; CLSM, confocal laser scanning microscopy.

The inhibitory effects of PT22 on biofilms were confirmed visually by SEM and CLSM images. The control group formed thick dense and heterogeneous masses while being treated with PT22, and the biofilms became looser, scattered, and broken (**Figure 9A**). The CLSM results are consistent with the results by SEM (**Figure 9B**). The biofilms treated with PT22, gentamycin, piperacillin, gentamycin + PT22, and piperacillin + PT22 presented reductions in biofilm formation (**Figures 9C, E**). The quantity of viable cells and the extracellular substrate of biofilms treated with gentamycin + PT22 were significantly reduced compared with gentamycin alone (**Figures 9C** c, d, **9E** c, d). After being treated with piperacillin + PT22, the quantity of viable cells was significantly reduced, and the bacterial mitosis was blocked, resulting in the formation of filamentous polymers and contraction of the cytoplasm compared with piperacillin alone (**Figures 9C** e, f and **9E** e, f). The same results were observed in which biofilms treated with PT22, gentamycin, piperacillin, gentamycin + PT22, and piperacillin + PT22 presented a reduction in mature biofilm (**Figures 9D, F**). The results demonstrated that PT22 dramatically enhanced the bactericidal effects of gentamycin and piperacillin on *P. aeruginosa* PAO1.

### Cytotoxicity of PT22 *in vitro*

**Figure 10:**
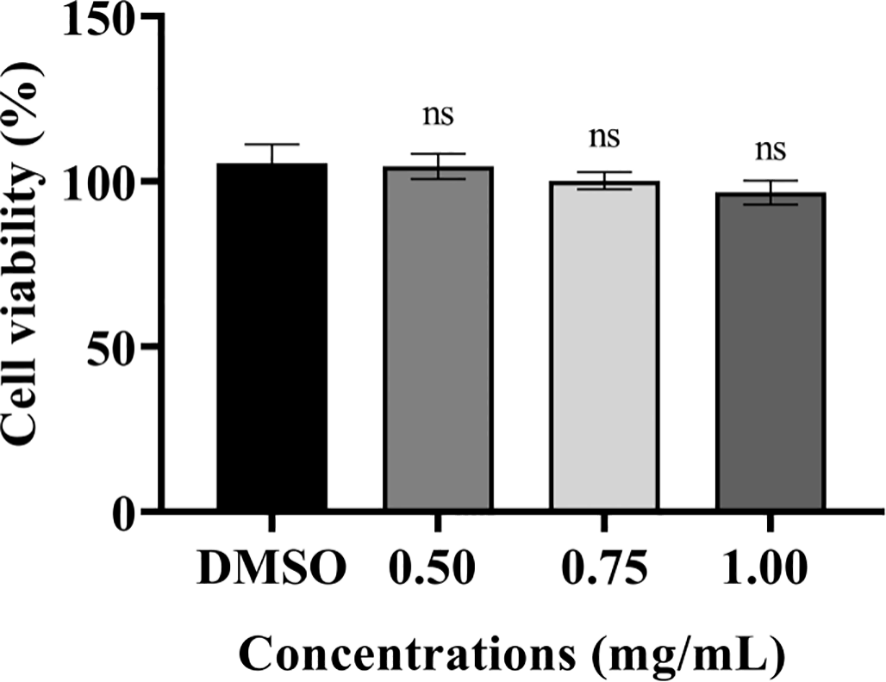
Cytotoxicity of PT22 *in vitro* on murine macrophages RAW 264.7 cells. DMSO was used as negative control. “ns” means no significance compared to the DMSO group by one-way ANOVA. DMSO, dimethyl sulfoxide.

The cytotoxic effects of PT22 were determined using cck-8 kit with murine macrophage RAW 264.7 cells. The cell viability was measured after 24-h incubation time. PT22 was observed to have cytotoxicity at their working concentrations (0.50 mg/mL, 0.75 mg/mL, and 1.00 mg/mL; **Figure 10**). This result indicates that PT22 could be further used for subsequent *in vivo* experiments.

### *G. mellonella* survival rates

**Figure 11:**
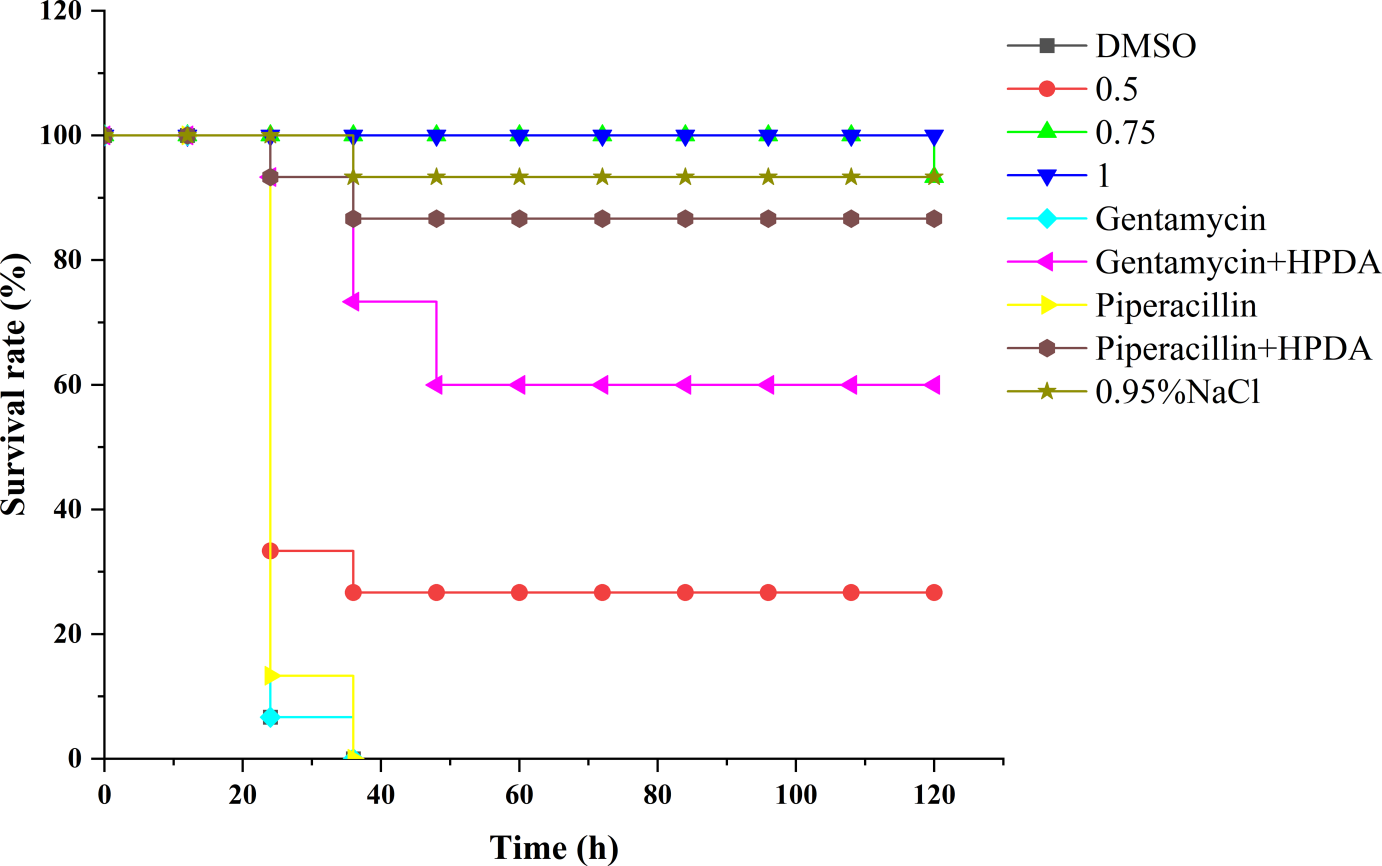
Survival rate of *Galleria mellonella* larvae infected by *Pseudomonas aeruginosa* PAO1 and recorded the survival rates of *G. mellonella* larvae in 120 h.

The *G. mellonella* larvae were used to evaluate the preservation effect of PT22, antibiotics, and the combination of PT22 and antibiotics on *P. aeruginosa* PAO1 infection (**Figure 11**). In the control group, gentamycin monotherapy group, and piperacillin monotherapy group, almost no survival rates were observed in *P. aeruginosa* PAO1 infected within 36 h. However, the infected larvae treated with PT22 (0.50 mg/mL, 0.75 mg/mL, and 1.00 mg/mL) for 120 h maintained survival rates of 26.67%, 93.33%, and 100%, respectively (**Figure 10**). For the gentamycin (4.00 μg/mL) + PT22 (0.50 mg/mL), the survival rate for infected larvae was 60.00% for 120 h. The survival rate for infected larvae was 86.67% after exposure to the piperacillin (4.00 μg/mL) + PT22 (0.50 mg/mL) for 120 h. The results suggested that PT22 could increase the survival rates of *G. mellonella* larvae infected by *P. aeruginosa* PAO1. The gentamycin combined with PT22 and piperacillin combined with PT22 maintained a survival rate of ≥ 60% and presented a higher survival rate than gentamycin and piperacillin monotherapy.

## Discussion

Over the past decades, extensive use and even abuse/misuse of antibiotics have resulted in the emergence of various multidrug-resistant (MDR) strains, including *P. aeruginosa* (Hazlett et al., 2019; Zhang et al., 2021). Troublesomely, the formation of biofilms preserves them from antibiotics, resulting in multidrug resistance (Tan et al., 2015). *P. aeruginosa* utilizes QS to regulate cell-to-cell communication and biofilm formation (Mukherjee and Bassler, 2019). Thus, inhibiting QS systems should be new targets for alternative or complementary treatments to conventional antibiotics (Hazlett et al., 2019). According to previous reports, meta-bromo-thiolactone (mBTL) inhibits the QS-related production of pyocyanin and biofilm formation, leading to the preservation of *Caenorhabditis elegans* and human lung epithelial cells from killing by *P. aeruginosa* (O Loughlin et al., 2013). Therefore, inhibiting the QS system is expected to be a potent strategy for avoiding MDR to *P. aeruginosa*.

Natural product chemistry is one endless frontier in the QSI field. Endophytic fungi are an under-explored source of bioactive natural products for the discovery of novel QSIs. Researchers have turned their attention to endophytes residing in plants to find new QSIs (Mookherjee et al., 2018). The crude extracts of an endophytic fungus, *Alternaria alternata* isolated from *Carica papaya*, possessed anti-QS properties against *P. aeruginosa* (Rashmi et al., 2018). Two pyran derivatives from the endophytic fungus *Lasiodiplodia venezuelensis* isolated from *Astrocaryum sciophilum* were purified as QSIs (Pellissier et al., 2021). In our group, 1-(4-amino-2-hydroxyphenyl)ethanone from endophytic fungus *P. liquidambari* S47 isolated from *Punica granatum* showed QS inhibitory activity against *P. aeruginosa* (Zhou et al., 2021). We also found that actinomycin D from endophyte *S. cyaneochromogenes* RC1 isolated from *A. catechu* L. presented great QSI activity against *P. aeruginosa* PAO1 (Zhou et al., 2018a). Furthermore, in the present work, we found that one alkaloid PT22 (1H-pyrrole-2,5-dicarboxylic acid) from the endophytic fungus *P. tephropora* FF2 isolated from *A. catechu* L. could inhibit the quorum sensing signaling molecules, virulence factor production, biofilm formation, motility and expression of QS-related genes, and function as an antibiotic accelerant against *P. aeruginosa* PAO1 infection. A previous study suggested that PT22 could inhibit IL-1β and TNF-α at 10 μM and 20 μM, respectively (Liu et al., 2024). This is the first report for PT22 as a QSI isolated from *A. catechu* L. endophytic fungus.

*P. tephropora* has not been isolated from *A. catechu* L. before. Although some species in the genus *Perenniporia* are mostly isolated as wood-inhabiting fungi, there are multiple bioactivities in secondary metabolites of these endophytes (Evans and Holmes, 2003; Pinruan et al., 2010; Wu et al., 2013). However, to our knowledge, no QSI secondary metabolites were reported except laccase from *P. tephropora* (Ben et al., 2007). The present study showed that PT22 isolated from *P. tephropora* FF2 impacts the QS of *P. aeruginosa* PAO1 without suppressing the proliferation at sub-MIC, as is evident from the growth curve analysis (**Figure 2**). Noticeably, there is significant depletion in the production of auto-inducers 3-oxo-C12-HSL and C4-HSL, which are reduced by 11.55% and 44.12%, respectively, after being treated with PT22 (1.00 mg/mL). It corresponds to the 70.41% and 83.56% reduction of encoded genes *las*I and *rhl*I, respectively. In addition, the *las*R and *rhl*R genes are reduced by 78.18% and 77.39%, respectively. The results indicated that PT22 may impede acylated homoserine lactone (AHL) production, resulting in QSI activity, and presents higher activity in reducing C4-HSL production. The endophytic fungus *Bruguiera gymnorhiza* purified fraction (BG138) interferes with the *P. aeruginosa* QS system, resulting in a decrease in QS signaling molecules and transcriptional levels of the QS-related genes (Dahibhate et al., 2022). Similar results were found in which citrinin could downregulate the QS-related genes (*las*I, *las*R, *rhl*I, and *rhl*R) and showed a QSI activity of *P. aeruginosa* (Ji et al., 2023).

Several QS-regulated virulence factors, such as pyocyanin, alginate, and rhamnolipid, were inhibited after the treatment of PT22. The reductions in *las*I/R and *rhl*I/R expressions after being treated with PT22 were correlated by assessing the activity of *las*-regulated elastase and protease and the production of *rhl*-regulated pyocyanin and rhamnolipid. Pyocyanin, one of the pigmented phenazine compounds that relate to host immune response evasion, is a signaling for gene regulation and sustaining the fitness of bacterial cells (Zeng et al., 2020). After exposure to PT22 (1.00 mg/mL), pyocyanin production was reduced by 73.05% (**Figure 4A**). It appears that the decrease of pyocyanin was also mediated by the downregulation of *phz*M, which was reduced by 86.08% (Jayaseelan et al., 2014). At sub-MIC (1.00 mg/mL), PT22 caused a significant decrease in rhamnolipid (34.06%), which may correspond to the decrease of *rhl*A (62.16%). The results suggested that PT22 could inhibit QS-regulated virulence factors of *P. aeruginosa* PAO1.

Forming biofilms is the key step to chronic infections of *P. aeruginosa*. The biofilms mainly consist of exopolysaccharides (EPSs), such as alginate, Pel, and Psl, and extracellular DNA (eDNA) (Petrova et al., 2011). Alginate is an essential virulence factor of *P. aeruginosa* because excessive production results in the clinically relevant mucoid phenotype (Ramsey and Wozniak, 2005). After exposure to PT22 (1.00 mg/mL), alginate production was reduced by 42.02% (**Figure 4E**), which corresponds to an 88.71% reduction of *alg*D. In addition, the Pel and Psl encoding genes *pel*A and *psl*A were reduced by 90.00% and 84.41%, respectively (**Figure 6**). The quantitative assay of biofilm result indicated that biofilm was approximately decreased by 64.74%. The inhibitory effects of PT22 on biofilms were confirmed visually by SEM and CLSM images. The results indicated that PT22 could inhibit QS-related biofilm formation of *P. aeruginosa* PAO1.

Motility is important in the biofilm formation of planktonic bacteria onto biotic and abiotic surfaces, which are responsible for chronic infections caused by *P. aeruginosa*. Therefore, inhibiting motility properties may be a highly potential strategy for controlling the biofilm formation of *P. aeruginosa* (Khan et al., 2020). Motility depends on the flagellum and the type IV pili in *P. aeruginosa*, which promote biofilm formation. Flagella encoded by *fil*C propel the bacteria in liquid (swimming motility) and semi-solid surfaces (swarming motility) by hydrodynamic forces. The type IVa pili encoded by *pil*A is responsible for twitching motility on solid surfaces (Burrows, 2012). After being treated with PT22 (1.00 mg/mL), swimming, swarming, and twitching were reduced by 90.73%, 86.11%, and 73.65%, respectively, which corresponded to the downregulation of *fil*C and *pil*A. The results demonstrated that PT22 could inhibit the QS-regulated motility of *P. aeruginosa* PAO1.

The production of enzymes and toxins is a major pathogenicity strategy of *P. aeruginosa*, which induces cytotoxicity and cell death in the host (Wood et al., 2023). ExoS is a toxin effector secreted by T3SS in *P. aeruginosa*. The adenosine diphosphate ribotransferase (ADRT) domain of ExoS targets various host proteins, which induces adverse effects on host cells, for instance, inhibition of DNA synthesis, vesicular trafficking and endocytosis, and cell death (Hauser, 2009). ExoY is anther T3SS-secreted toxin effector, which results in a disruption in the actin cytoskeleton and the increase of endothelial permeability (Sánchez-Jiménez et al., 2023). The *exo*S and *exo*Y genes were downregulated by 88.99% and 89.99%, respectively. The results showed that PT22 may inhibit the T3SS-secreted toxins of *P. aeruginosa* PAO1. In addition, it is reported that various TCSs regulate the virulence factors of *P. aeruginosa* (Francis et al., 2017). We found that the expression level of *gac*A related to the GacS/GacA TCS, which is a master regulator of virulence, swarming motility, and biofilm formation, was reduced by 68.14%. Although there are two types of regulation systems, TCS and QS in *P. aeruginosa*, which regulate the expression of virulence factors, the relationship or interaction between them needs to be further investigated.

As antibiotics rapidly lose their efficacy, alternative strategies are urgently needed. Synergistic enhancement of antibiotics and sub-MIC QSI is a potentially exciting alternative strategy. Mishra et al (Mishra et al., 2020). reported that 2,4-di-*tert*-butylphenol from an endophytic fungus, *Daldinia eschscholtzii*, possesses QSI activity and presents synergism with ampicillin to kill *P. aeruginosa*. After exposure to gentamycin + PT22 and piperacillin + PT22, the biomass and viable cells in biofilms were significantly reduced compared with those exposed to gentamycin and piperacillin alone, as well as presented a higher survival rate (≥60%) of *G. mellonella* larvae infected by *P. aeruginosa* PAO1 than gentamycin or piperacillin alone. The results indicated that PT22 extremely enhanced the bactericidal effects of gentamycin or piperacillin on *P. aeruginosa* PAO1. Similar research revealed that the QSI resveratrol may act as a potential accelerant of aminoglycoside to enhance the antibiotic sensitivity of *P. aeruginosa* (Zhou et al., 2018a). PT22 combined with gentamycin or piperacillin could inhibit the biofilm formation and erase the mature biofilm, resulting in increased antibiotic sensitivity of *P. aeruginosa* PAO1 and the survival rate of *G. mellonella* larvae infected by *P. aeruginosa* PAO1.

Molecular docking analysis of receptors with their ligands was performed as binding rigidly to the receptors. The strong interaction between the receptors and the ligands may be due to the binding of specific sites, which makes the conformational changes of the receptor, suggesting that it may act as a potential inhibitor of this protein. PT22 could bind to LasI with the same Arg30 residue and approximately equal affinity energy compared to 3-oxo-C12-HSL, indicating that PT22 may repress the synthesis of 3-oxo-C12-HSL. PT22 could bind to RhlI with the same Val138 residue, suggesting that PT22 also represses the synthesis of C4-HSL. PT22 could bind to LasR with the same Tyr56 and Asp73 residues compared to 3-oxo-C12-HSL, while Ser135 and Asp81 residues are the same residues binding to RhlR between PT22 and C4-HSL. Interestingly, the interaction docking energy between RhlR and C4-HSL is −4.4 kcal/mol, and that between RhlR and PT22 is −6.1 kcal/mol. PT22 required less energy for docking with RhlR, indicating that PT22 exhibits better affinity for RhlR than C4-HSL. The results indicated that PT22 inhibited QS probably through competing with AHL for the binding sites of receptors, especially RhlR, which corresponds with the reduction of AHL production. In the earlier stage of bacterial reproduction, PT22 binds to signaling molecule secreted proteins and occupies key sites involved in signaling molecule synthesis. Additionally, PT22 could bind to MDR efflux pump-related protein MexB (**Figure 7F**), but its affinity energy is lower than that of gentamycin and piperacillin (**Supplementary Table S3**). Gentamycin presents a more significant reduction in inhibition of biofilm formation and eradication of mature biofilm than piperacillin (**Figures 8C, E**). However, after being treated with gentamycin + PT22 and piperacillin + PT22, although both of them present more remarkable depletion compared with gentamycin or piperacillin alone, there is no significance between gentamycin + PT22 and piperacillin + PT22, indicating that PT22 presents more significant accelerating effect on piperacillin rather than gentamycin. It appears that PT22 and piperacillin have similar binding pocket sites, which bind to MexB via the same covalent bonds at Thr115 (**Figure 7F**). After the combination of PT22 and piperacillin, the efflux pump is suppressed. Then, piperacillin entered and accumulated intracellularly easily. The combination of PT22 and piperacillin could increase the antibiotic sensitivity of *P. aeruginosa* PAO1 and the survival rate of *G. mellonella* larvae infected by *P. aeruginosa* PAO1 compared to PT22 combined with gentamycin (**Figure 11**). The results demonstrated that PT22 exhibited significant QSI activity and acted as an antibiotic accelerant against *P. aeruginosa* PAO1 infection.

Additionally, we also investigated the cytotoxicity of PT22 on macrophages RAW 264.7 cells. The cytotoxicity of the compounds is important if they are to be used in animal studies for subsequent drug development. Results indicated that PT22 has no cytotoxicity. The same results suggest that itaconimides as a QSI against *P. aeruginosa* are not toxic up to 40-µM concentration (Fong et al., 2019). Previous studies have shown that mice treated with QSI afford better survival profiles and lower bacterial count loads when compared to the control (Jakobsen et al., 2013). Still, further studies are needed to qualify the efficacy of PT22 in mouse models for their potential as anti-biofilm agents, as well as their pharmacodynamic and pharmacokinetic profiles.

## Conclusion

1H-Pyrrole-2,5-dicarboxylic acid, first isolated from *P. tephropora* FF2, the endophytic fungus of *A. catechu* L., could inhibit virulence factors and biofilm formation of *P. aeruginosa* PAO1. It exhibits potent QSI activity and functions as an antibiotic accelerant against *P. aeruginosa* PAO1 infection.

## Data availability statement

The datasets presented in this study can be found in online repositories. The names of the repository/repositories and accession number(s) can be found below: https://www.ncbi.nlm.nih.gov/genbank/, OR349622.

## Ethics statement

The manuscript presents research on animals that do not require ethical approval for their study.

## Author contributions

JL: Methodology, Resources, Software, Visualization, Writing – original draft. ZW: Conceptualization, Project administration, Software, Writing – review & editing. YZ: Methodology, Resources, Software, Writing – original draft. WW: Methodology, Software, Visualization, Writing – original draft. AJ: Funding acquisition, Project administration, Supervision, Writing – review & editing. ST: Methodology, Project administration, Software, Supervision, Writing – review & editing.
