# Supplementary material for: 1H-Pyrrole-2,5-dicarboxylic acid, a quorum sensing inhibitor from one endophytic fungus in Areca catechu L., acts as antibiotic accelerant against Pseudomonas aeruginosa
Source: Front Cell Infect Microbiol. 2024 Jul 2;14:1413728. doi: 10.3389/fcimb.2024.1413728 (PMC11250523; doi:10.3389/fcimb.2024.1413728)
Supplement: Supplementary file 1 [file DataSheet_1.pdf]

Supplemental information

Table S1 Minimum inhibitory concentrations (MICs) of PT22 and antibiotics against *P. aeruginosa* PAO1.

Agents	MICs（μg/mL）
PT22	> 2000
gentamycin	8
piperacillin	8

Table S2 PCR primers for real-time quantitative PCR (RT-qPCR).

genes	Primer directions	Primer sequences (5'-3')
*las*I	Forward	GGCTGGGACGTTAGTGTCAT
	Reverse	AAAACCTGGGCTTCAGGAGT
*las*R	Forward	ACGCTCAAGTGGAAAATTGG
	Reverse	TCGTAGTCCTGGCTGTCCTT
*rhl*I	Forward	AAGGACGTCTTCGCCTACCT
	Reverse	GCAGGCTGGACCAGAATATC
*rhl*R	Forward	CATCCGATGCTGATGTCCAACC
	Reverse	ATGATGGCGATTTCCCCGGAAC
*pqs*R	Forward	AACATGTTCCTCCAGGTCAT
	Reverse	GTTGAGATTGAAGGCGATGT
*las*A	Forward	GCCGCTGAATGACGACCTGT
	Reverse	TCAGGGTCAGCAACACTT
*las*B	Forward	AAGGCCTTGCGGGTATCC
	Reverse	CGTGTACAACCGTGCGTTCT
*rhl*A	Forward	CCAAGGACGACGAGGTGGA
	Reverse	CGAGCATCGCCTGGTTCA
*phz*R	Forward	CTACCTTCGGCGACCTG
	Reverse	CCTTGTTGCTGGAGTTGA
*phz*M	Forward	GAATGGAAGTCCCGTTGC
	Reverse	GCCCTCGACATCCCTCA
*pel*A	Forward	GGA ACA GCC AGG TAA TGG AC
	Reverse	TCC AGG GTA TCG AGG AAC AG
*alg*D	Forward	CTGCCTGCCCAAGGATGT
	Reverse	GTCGTGGCTGGTGATGAGAT
*psl*A	Forward	CGGTCAGCGAATACAGCTC
	Reverse	TTGATCTTGTGCAGGGTGTC
*exo*S	Forward	TCAGGTACCCGGCATTCACTACGCGG
	Reverse	CACTGCAGGTTCGTGACGTCTTTCTTTTA

*exo*Y	Forward	TCCAAGCTTATGCGTATCGACGGTCATC
	Reverse	CGTATCGATCCGAGGGGGGTGTATCTGACC
*tox*A	Forward	CCCGGCGAAGCATGAC
	Reverse	GGGAAATGCAGGCGATGA
*fli*C	Forward	CGACAAGGGTGTACTGACCA
	Reverse	GACCTTCACTGCGACCTGAC
*pil*A	Forward	GCGACAGCGACTCTTCAAC
	Reverse	CGGTATCCTGGCGGCAATT
*mex*B	Forward	GTGTTCGGCTCGCAGTACTC
	Reverse	AACCGTCGGGATTGACCTTG
*gac*A	Forward	GTCGTGGTAGTCACCGTCTG
	Reverse	CGAATCGAAGGGGGAATCGT
*rps*L	Forward	GCAACTATCAACCAGCTGGTG
	Reverse	GCTGTGCTCTTGCAGGTTGTG

Table S3 Details of the docked complex of PT22 and autoinducers with their target receptors.

Receptors	Ligands	Hydrogen bonds	Hydrophobic bonds	Docking energy (kcal/mol)
LasI	3-oxo-C12-HSL	Arg30, Thr145	Phe117, Val26, Ile107, Ala106, Phe105, Val48, Val143, Arg104, Thr144	-6.4
	PT22	Arg104, Phe105, Val43, Arg30	Thr145, Phe27, Thr144, Thr145	-6.2
LasR	3-oxo-C12-HSL	Trp60, Asp73, Thr75, Ser129, Tyr56	Leu110, Leu36, Trp88, Tyr64, Tyr47, Ala50, Leu40, Leu125, Gly126, ALa127, Phe101	-8.6
	PT22	Tyr56, Tyr64, Asp73, Tyr93, Ser129	Trp60, Phe101, Ala105, Trp88, Thr75, Leu36	-7.1
RhlI	C4-HSL	Tyr105, Val138	Ala137, Phe147, Thr139, Leu80, Trp34, Arg104, Leu102, Ser103	-5.4
	PT22	Val138, Arg104	Ala137, Leu80, Trp34, Ser103, Tyr105, Met143, Thr139	-5.1
RhlR	C4-HSL	Tyr64, Ser135, Asp81, Trp68	Ala111, Phe101, Trp96, Ile84, Gly46, Tyr72	-4.4
	PT22	Asp81, Ser135	Val33, Tyr45, Ile84, Gly46, Val60, Tyr72, Tyr64, Trp96, Ala44	-6.1
PqsR	NHQ	-	Thr166, Lys167,	-7.1

				Ala168, Thr265, Ala102, Ile149, Pro238, Phe221, Pro129, Ala130, Leu197, Leu208, Ile236, Ala237	
	PT22	His204, Ile236, Gln194		Leu197, Leu208, Ala130, Met224, Ser196	-5.8

Table S4 Details of the docked complex of gentamycin, piperacillin, and PT22 with MexB.

Receptors	Ligands	Hydrogen bonds	Covalent bonds	Hydrophobic bonds	Docking energy (kcal/mol)
MexB	Gentamycin	Phe617, Asn718, Thr93, Tyr77, Ser79	-	Lys134, Ala42, Glu673, Pro40, Gly860, Thr91, Glu81, Phe683, Ser80, Glu816, Lys814	-8.5
	Piperacillin	-	Thr115, Val64, Gln63, Met69, Ile127	Gln58, Glu66, Val92, Ile78, Ile90, Gln68, Leu111, Ala114, Gln123	-8.6
	PT22	-	Leu111, Thr115	Ile65, Gln123, Ile127, Leu118, Ala114, Gln112	-5.9

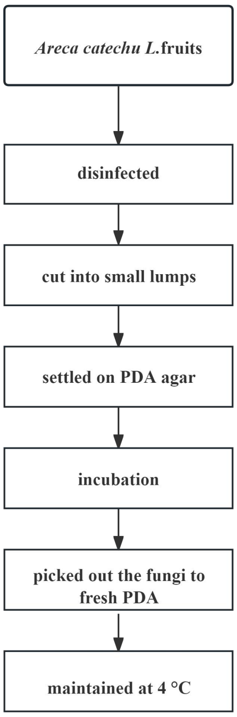

Fig. S1 The isolation of endophytic fungi strains workflow.

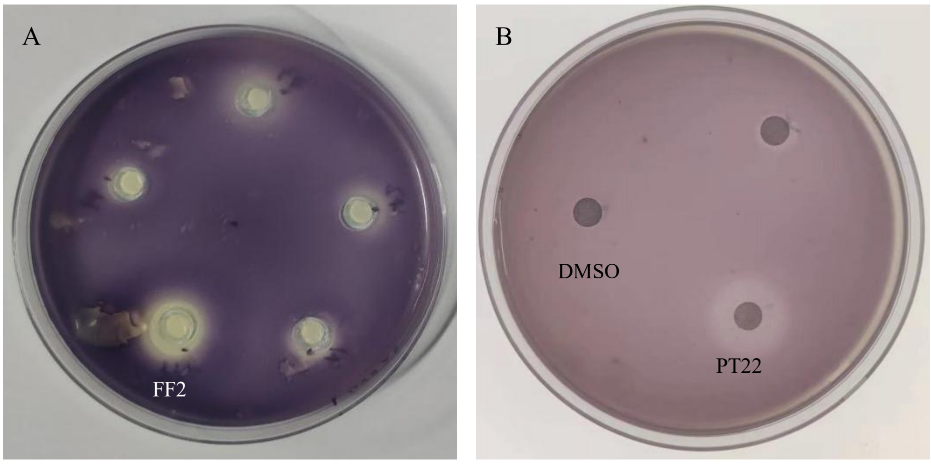

Fig. S2 The activities of secondary metabolites from endophytic fungi of *Areca catechu* L. with a biosensor strains *C. violaceum* CV026. DMSO was used as a negative control. (A) secondary metabolites from endophytic fungi. (B) secondary metabolites from *P. tephropora* FF2.

A

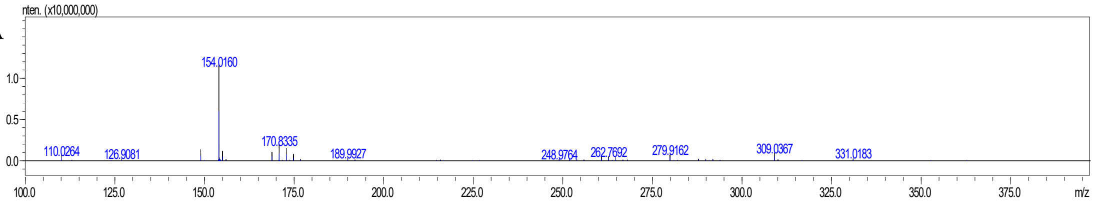

B

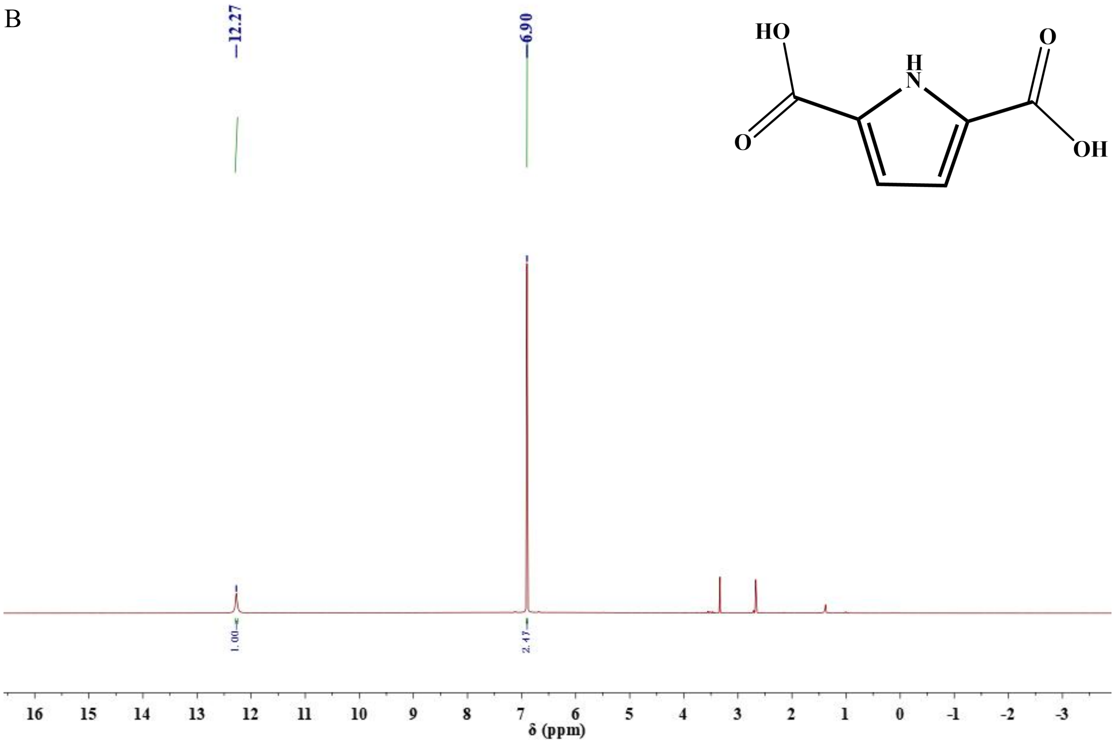
12.27
6.90
HO
O
H
N
O
OH
1.00
2.47
16 15 14 13 12 11 10 9 8 7 6 5 4 3 2 1 0 -1 -2 -3
δ (ppm)

C

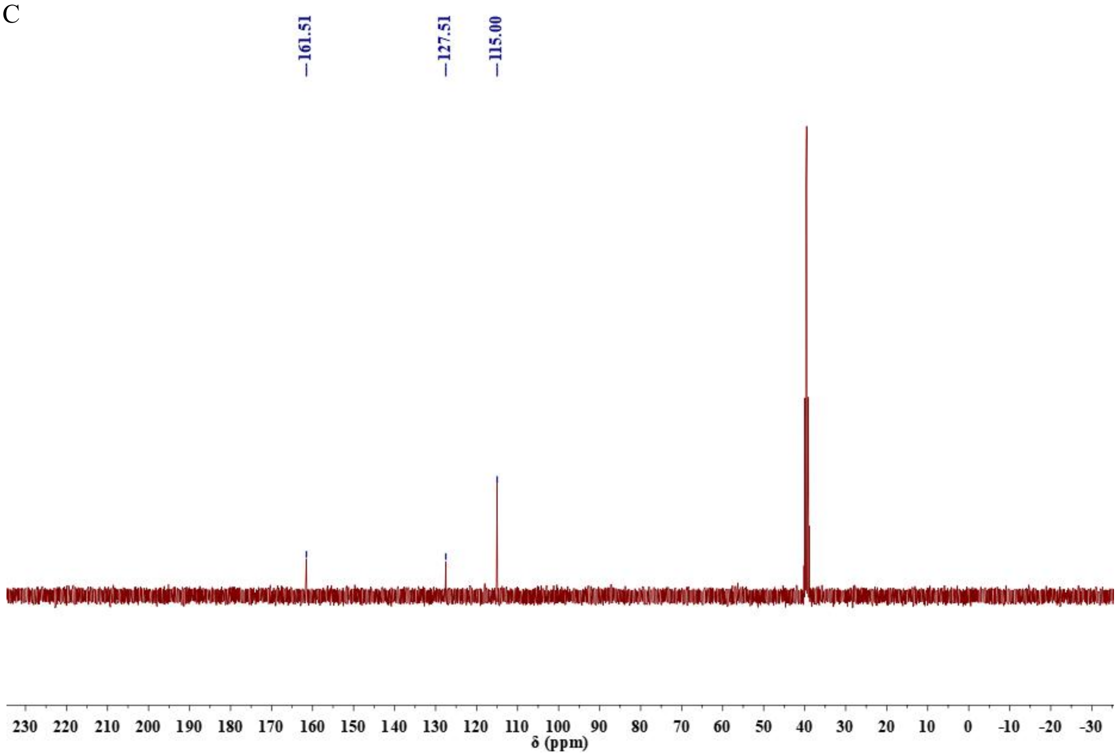
161.51
127.51
115.00
230 220 210 200 190 180 170 160 150 140 130 120 110 100 90 80 70 60 50 40 30 20 10 0 -10 -20 -30
δ (ppm)

D

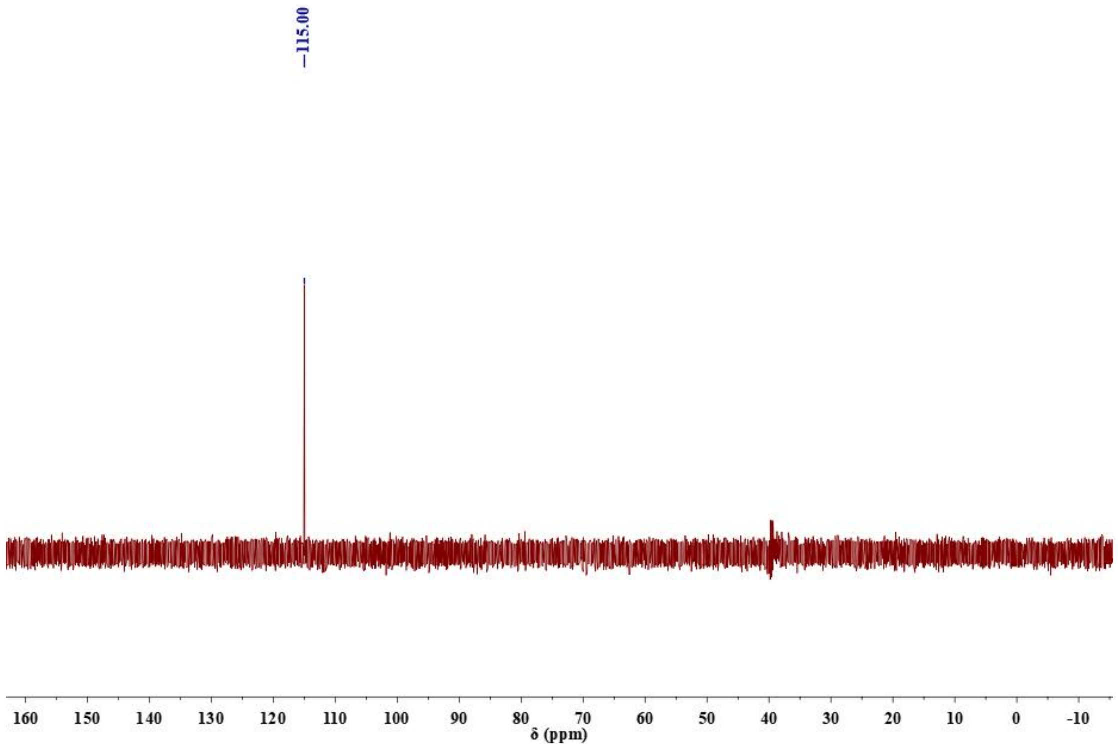

Fig. S3 The HR-ESI-MS (A) and NMR spectra of PT22 (B, C, and D). ^{1}H NMR (DMSO-d_6, 400MHz): 12.27 (1H, brs, NH), 6.90 (2H, d, J = 2.5Hz, H-3, H-4). ^{13}C NMR (DMSO-d_6, 100MHz): 161.51 (2 × COOH), 127.51 (C-2, C-5), 115.00 (C-3, C-4). EIMS (70 eV) m/z (rel. int.): $[M]^{-}$ 154.0160,calcd for C6H5NO4, 155.1082.

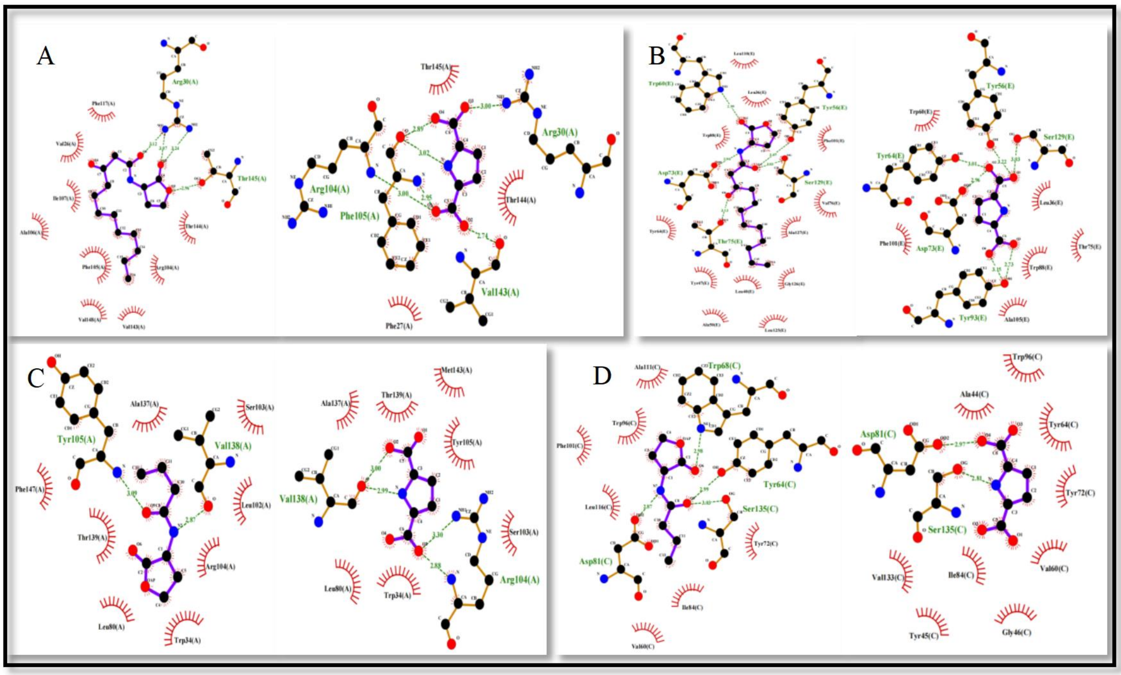

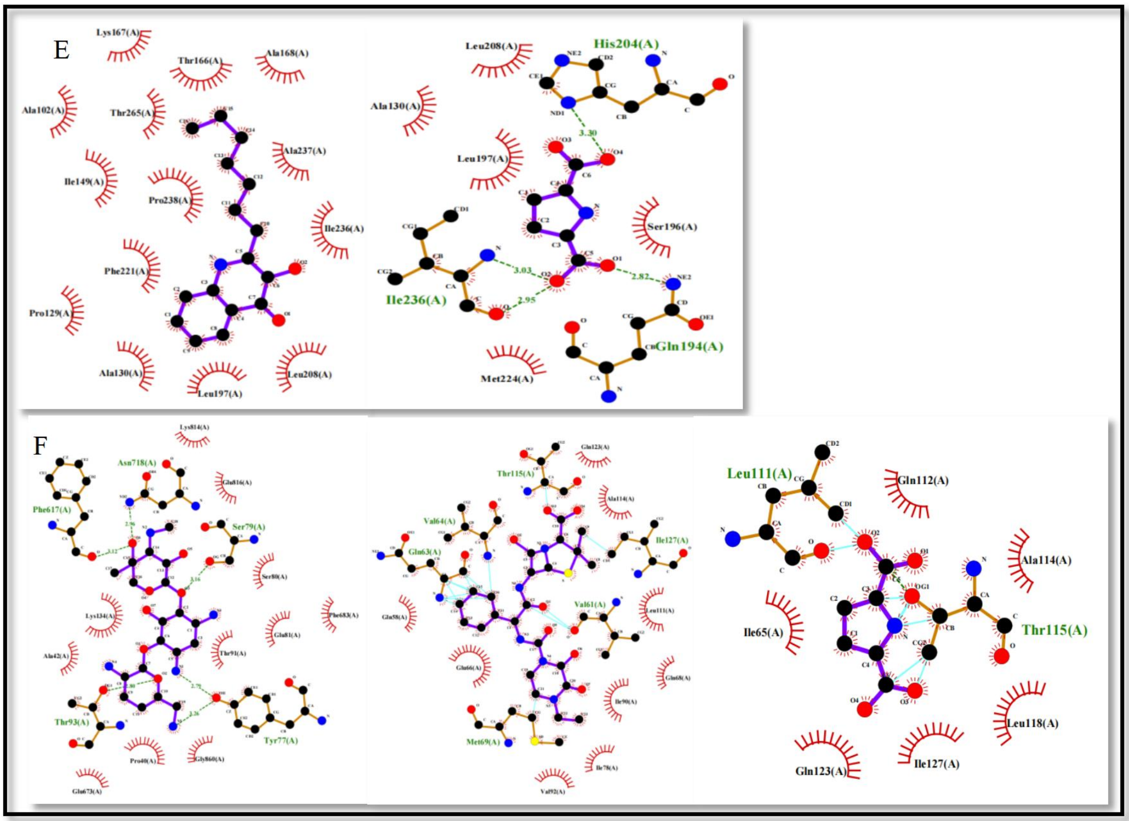

Fig. S4 Interactions between QS receptor proteins and various ligands. (A) LasI bound to 3-oxo-C12-HSL and PT22; (B) LasR bound to 3-oxo-C12-HSL and PT22; (C) RhlI bound to C4-HSL and PT22; (D) RhlR bound to C4-HSLand PT22; (E) PqsR bound to NHQ and PT22; (F) MexB bound to gentamycin, piperacillin, and PT22.
